# Australian Assassins, Part II: A review of the new assassin spider genus *Zephyrarchaea* (Araneae, Archaeidae) from southern Australia

**DOI:** 10.3897/zookeys.191.3070

**Published:** 2012-05-07

**Authors:** Michael G. Rix, Mark S. Harvey

**Affiliations:** 1 Department of Terrestrial Zoology, Western Australian Museum, Locked Bag 49, Welshpool DC, Perth, Western Australia 6986, Australia; 2 Research Associate, Division of Invertebrate Zoology, American Museum of Natural History, New York, NY 10024, USA; 3 Research Associate, California Academy of Sciences, 55 Music Concourse Drive, San Francisco, CA 94118, USA; 4 Adjunct Professor, School of Animal Biology, University of Western Australia, 35 Stirling Highway, Crawley, Perth, Western Australia 6009, Australia

**Keywords:** New species, taxonomy, conservation, cytochrome *c* oxidase, mitochondrial DNA, Palpimanoidea

## Abstract

The Assassin Spiders of the family Archaeidae from southern Australia are revised, with a new genus (*Zephyrarchaea* **gen. n.**) and nine new species described from temperate, mesic habitats in southern Victoria, South Australia and south-western Western Australia: *Zephyrarchaea austini* **sp. n.**, *Zephyrarchaea barrettae* **sp. n.**, *Zephyrarchaea grayi* **sp. n.**, *Zephyrarchaea janineae* **sp. n.**, *Zephyrarchaea marae* **sp. n.**, *Zephyrarchaea marki* **sp. n.**, *Zephyrarchaea melindae* **sp. n.**, *Zephyrarchaea porchi* **sp. n.** and *Zephyrarchaea vichickmani* **sp. n.** Specimens of the type species, *Zephyrarchaea mainae* (Platnick, 1991), **comb. n.**, are redescribed from the Albany region of Western Australia, along with the holotype female of *Zephyrarchaea robinsi* (Harvey, 2002) **comb. n.** from the Stirling Range National Park. The previously described species *Archaea hickmani* Butler, 1929 from Victoria is here recognised as a **nomen dubium**. A key to species and multi-locus molecular phylogeny complement the species-level taxonomy, with maps, habitat photos, natural history information and conservation assessments provided for all species.

## Introduction

**Figure 1.**
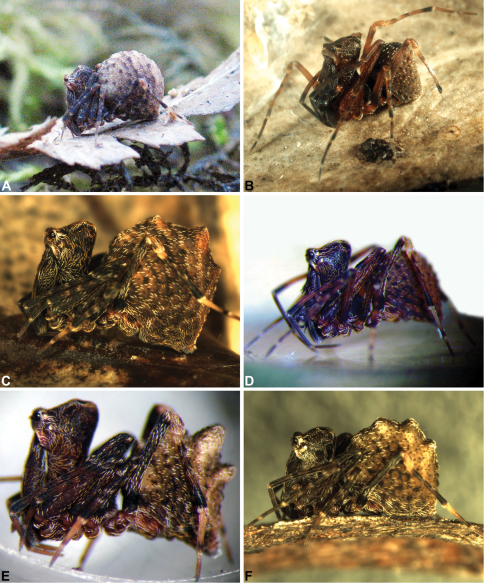
Habitus images of live Archaeidae from southern Australia: **A** paratype female *Zephyrarchaea vichickmani* sp. n. from Acheron Gap, Central Highlands, Victoria; **B** holotype male *Zephyrarchaea barrettae* sp. n. from Talyuberlup Peak, Stirling Range National Park, Western Australia; **C** paratype female *Zephyrarchaea janineae* sp. n. from Karri Valley, Western Australia; **D** male *Zephyrarchaea marki* sp. n. from Thistle Cove, Cape Le Grand National Park, Western Australia; **E** female *Zephyrarchaea mainae* (Platnick) from William Bay National Park, Western Australia; **F** female *Zephyrarchaea mainae* from Bremer Bay, Western Australia.

Once considered among the most enigmatic and poorly known of spider families, recent research into the assassin spiders of the family Archaeidae (Fig. 1) has revealed three diverse, highly endemic faunas from southern Africa, Madagascar and Australia, each of considerable evolutionary and conservation significance, and all the focus of modern revisionary systematic studies that have transformed our understanding of archaeid evolution and biogeography (see Platnick 1991a, 1991b, Lotz 1996, 2003, 2006, Harvey 2002a, Wood et al. 2007, Wood 2008, Rix and Harvey 2011, 2012). Although widely known from Mesozoic and Tertiary fossil deposits on multiple continents (see Dunlop et al. 2012), the Recent archaeid fauna consists of 54 described species in three genera (Platnick 2012), with numerous new species still to be described from African, Malagasy and Australian regions (H. Wood, pers. comm., M. Rix pers. obs.). These species, all remarkable for their araneophagic ecology and highly autapomorphic morphology, have highlighted the contrasting patterns of speciation and endemism that occur in Australian versus Old World taxa, and the importance of the unique archaeid carapace morphology in the evolution of the group (see Wood et al. 2007, Rix and Harvey 2012). The distinctive Australian fauna, while long neglected taxonomically and once presumed to be comparatively species-poor, has recently been shown to be far more widespread and species-rich than previously expected, thanks to dedicated field surveys and significant advances in our understanding of archaeid biology, ecology and biogeography (see Rix and Harvey 2011, 2012).

The history of the discovery and documentation of Archaeidae in Australia is one of significant recent, almost exponential progress, with over 85% of currently recognised species described in the past five years, and only four valid taxa described in the eight decades since the first species, *Austrarchaea hickmani* (Butler, 1929) was first recorded from Victoria. The second true archaeid to be recorded from Australia, *Austrarchaea nodosa* (Forster, 1956), was described from the Lamington Plateau, south-eastern Queensland, and nearly 30 years later Forster and Platnick (1984) described a third species, *Austrarchaea daviesae* Forster & Platnick, 1984, from the Wet Tropics of north-eastern Queensland, further erecting the new genus *Austrarchaea* to include all of the Australian taxa. Archaeidae were unknown from Western Australia until *Austrarchaea mainae* Platnick, 1991b (Figs 1E-F) was described from the Torndirrup Peninsula, near Albany (first reported by Dyer and Lyon 1983, Main 1987a, 1987b), and Main (1995) reported the discovery in the 1970s of six unidentified juvenile specimens from near Pemberton. The first archaeid specimen to be recorded from the Stirling Range National Park in southern Western Australia was collected in 1996 and this species, *Austrarchaea robinsi* Harvey, 2002a, remained the only assassin spider to have been collected in Western Australia for a further 10 years. In 2006, living adult specimens of *Zephyrarchaea janineae* sp. n. (Fig. 1C) were discovered in wet forest near Pemberton, catalysing the first of many such discoveries in Western Australia, and highlighting the importance of temperate coastal heathlands as a habitat for assassin spiders. Indeed, since the pioneering revisionary work of Forster and Platnick (1984), when only eight adult archaeids had been recorded for the whole of Australia, over 500 specimens of Archaeidae have since been found throughout Queensland, New South Wales, Victoria, South Australia and Western Australia, revealing a diverse Australian fauna, characterised by numerous new species and mostly allopatric, relictual, short-range endemic taxa (Harvey 2002b, Harvey et al. 2011, Rix and Harvey 2011, 2012).

**Figure 2.**
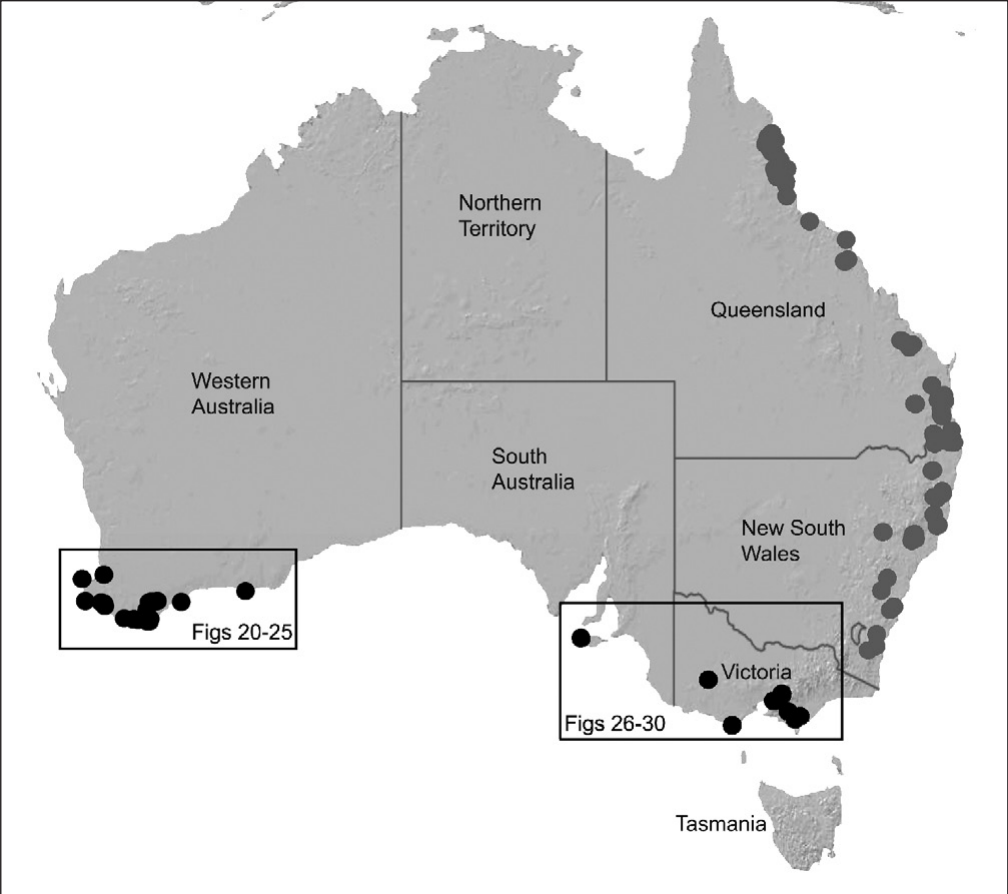
Map showing the known distribution of Archaeidae in Australia, with locality records for species of *Zephyrarchaea* highlighted in black. Note the disjunct distribution of the genus in south-eastern and south-western mainland Australia.

**Figure 3.**
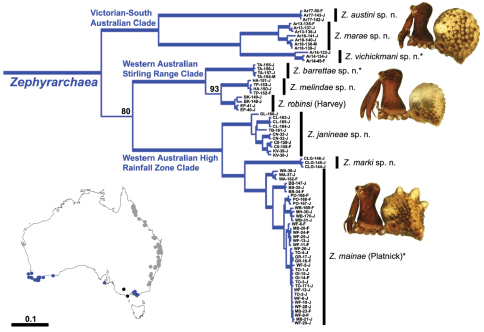
Phylogeny of *Zephyrarchaea* species from Rix and Harvey (2012), showing results from a combined, gene-partitioned Bayesian analysis of that study’s multi-gene dataset (2591 bp: COI, COII, tRNA-K, tRNA-D, ATP8, ATP6, H3). Clades with >95% posterior probability support are denoted by thickened branches, with lower individual clade support values shown above nodes. Note the presence of three main regional clades, each illustrated with an exemplar species (highlighted*). The Victorian species *Zephyrarchaea grayi* sp. n. and *Zephyrarchaea porchi* sp. n. were not able to be sequenced for this study (shown as black dots on inset map).

**Figure 4.**
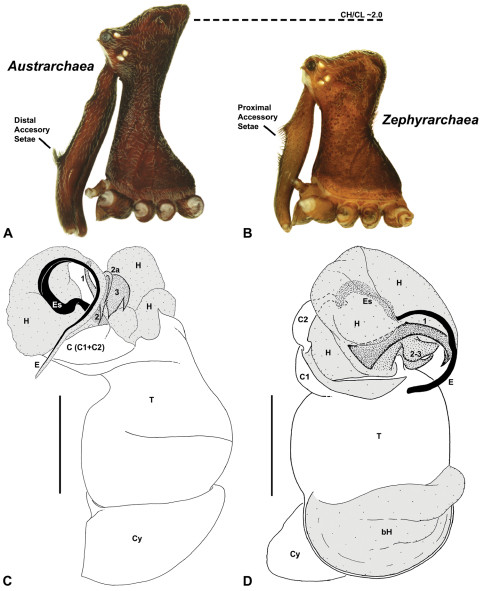
Diagnostic characters of *Zephyrarchaea* gen. n. and *Austrarchaea* Forster & Platnick. **A–B**, Cephalothorax, lateral view, showing differences in carapace height and the position of accessory setae on male chelicerae: **A**, male *Austrarchaea harmsi* Rix & Harvey; **B**, male *Zephyrarchaea marki* sp. n. **C–D**, Expanded male pedipalps, retro-ventral view, showing differences in the articulation and fusion of the conductor sclerites: **C**, *Austrarchaea helenae* Rix & Harvey; **D**, *Zephyrarchaea marae* sp. n. bH = basal haematodocha; C = conductor; C1–2 = conductor sclerites 1–2; Cy = cymbium; E = embolus; Es = embolic sclerite; H = distal haematodocha; T = tegulum; (TS)1–3 = tegular sclerites 1–3. Scale bars: C–D = 0.2 mm.<br/>

The current paper – the second in a series revising the Archaeidae of Australia – presents a taxonomic revision of the assassin spiders from temperate ‘southern Australia’, including those species from Victoria, South Australia and south-western Western Australia (Fig. 2). A new genus, *Zephyrarchaea* (Figs 1, 4), is described to include the type species, *Zephyrarchaea mainae*, along with *Zephyrarchaea robinsi* and nine new species. These taxa were found to form a monophyletic and highly divergent clade in a recent molecular phylogenetic analysis (Rix and Harvey 2012; Fig. 3), sister to all other species of Archaeidae from eastern Australia. This revision takes the total number of described Australian Archaeidae to 30 species, with the remaining, unrevised archaeids from north-eastern Queensland to be described in the third and final monograph of this series.

## Material and methods

All taxa were described and illustrated from specimens stored in 75% or 95% ethanol. Digital images were taken using a Leica MZ16A binocular microscope and a Leica DM2500 compound microscope, with auto-montage images captured using Leica DFC500 mounted cameras with Leica Application Suite Version 3.6.0 software. Male left pedipalps were dissected prior to imaging and bulbs were aligned for standardised comparison in the retrolateral and prolateral positions illustrated; expanded pedipalps were illustrated in a retro-ventral position. Female genitalia were dissected and cleared in a 10% lactic acid plus 90% glycerol solution, prior to mounting on temporary glass slides. Illustrations were made on Utoplex tracing paper, using printed template auto-montage images. Maps were generated using ArcMap version 9.3.1 (ESRI Inc.) with Virtual Earth (Microsoft Corp.).

Measurements are in millimetres (rounded to the nearest hundredth of a millimetre) and were taken using an ocular graticule on a Leica M80 binocular microscope. Left legs were removed from specimens prior to taking measurements and imaging lateral body profiles. Lateral profile images were standardised for inter-specific comparison by vertically aligning the centre of each left anterior median eye with the lower anterior margin of the carapace (above the labrum) (Fig. 8A). Carapace height was measured in lateral view, from the margin of the pars thoracica above coxa II to the highest point of the pars cephalica (Fig. 7). Carapace length was measured from the lower anterior margin of the carapace (above the labrum) to the posterior margin of the pars thoracica (above the pedicel) (Fig. 7). ‘Neck’ width was measured in lateral view, at the narrowest point of the carapace, with total length, carapace width, abdomen length and abdomen width all measured in dorsal view. To quantify inter-specific variation in the shape of the cephalothorax and ‘head’, three morphometric ratios (the *carapace height to carapace length* [CH/CL] *ratio*; the *post-ocular ratio* [P.O. ratio]; and the ratio of *highest point of pars cephalica* [HPC] *to post-ocular length ratio*) were derived from lateral profile images (Figs 7-9) as defined and discussed by Rix and Harvey (2011) (see also Fig. 8A).

### Conventions

Specimens sequenced for the molecular analysis of Rix and Harvey (2012) are denoted by superscript codes, which correspond to specimen codes as shown in Rix and Harvey (2012, table 1, fig. 4). For species diagnoses, molecular autapomorphies for mitochondrial cytochrome oxidase genes (COI-COII; see Harvey et al. 2008, Cook et al. 2010, Rix and Harvey 2011) are coded according their nucleotide number (1-1609), as defined in Rix and Harvey (2011, table 3).

### Abbreviations used in the text are as follows:

AME Anterior median eye/s

C1-2 Conductor sclerites 1–2

CH/CL Carapace height (CH) to carapace length (CL) ratio

F1/CL Femur I length (F1) to carapace length (CL) ratio

HPC Highest point of pars cephalica

HT 1–6 Abdominal hump-like tubercles 1–6

PME Posterior median eye/s

TS 1–3 Tegular sclerites 1–3

### Specimens described in this study are lodged at the following institutions:

AMNH American Museum of Natural History, New York (N. Platnick, L. Sorkin)

AMS Australian Museum, Sydney (G. Milledge)

CASENT California Academy of Sciences, San Francisco (C. Griswold, A. Carmichael)

MV Museum Victoria, Melbourne (P. Lillywhite)

QMB Queensland Museum, Brisbane (R. Raven, O. Seeman)

SAM South Australian Museum, Adelaide (L. Chisholm)

WAM Western Australian Museum, Perth (MSH, J. Waldock)

## Taxonomy

### Family Archaeidae Koch & Berendt, 1854

#### Genus Zephyrarchaea

Rix & Harvey gen. n.

urn:lsid:zoobank.org:act:BEE3BD64-0A61-40D4-880A-2A562847A855

http://species-id.net/wiki/Zephyrarchaea

##### Type species.

*Austrarchaea mainae* Platnick, 1991b.

##### Etymology.

The generic name is derived from the Latin ‘zephyrus’, meaning ‘west wind’ (Brown 1956), in reference to the diversity of this genus in south-western Australia, and the windy, coastal habitats occupied by several species, including the type species.

##### Diagnosis.

Species of *Zephyrarchaea* can be distinguished from all eastern Australian species of *Austrarchaea* by the significantly shorter carapace (CH/CL ratio << 2.0) (Fig. 4B cf. Fig. 4A, 7), by the presence of accessory setae on or adjacent to the proximal (rather than the distal) bulge of the male cheliceral paturon (Fig. 4B cf. Fig. 4A), and by the shape of the two conductor sclerites on the male pedipalp, which are hinged, unfused and moveable (Fig. 4D cf. Fig. 4C), together forming a fully articulated cradle for the unexpanded embolus (Fig. 10E). Like species of *Austrarchaea*, the genus can be further distinguished from Malagasy and African species of *Eriauchenius* and *Afrarchaea* by the presence of numerous, clustered spermathecae in females (Fig. 16G) and by the presence of a long, wiry embolus on the pedipalp of males (Figs 10E, 16E) (Forster and Platnick 1984, Wood 2008, Rix and Harvey 2011).

##### Description.

Small, haplogyne, araneomorph spiders; total length 2.5 to 4.5.

**Figure 5.**
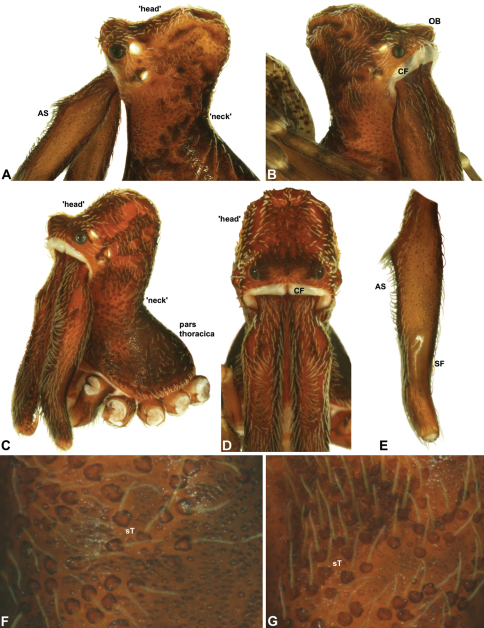
Carapace morphology of *Zephyrarchaea* species. **A–B**, *Zephyrarchaea mainae* (Platnick): **A**, male pars cephalica, dorso-lateral view, showing accessory setae (AS) on and adjacent to proximal cheliceral bulge; **B**, female pars cephalica, antero-lateral view, showing cheliceral foramen (CF) and ocular bulge (OB). **C–D**, *Zephyrarchaea marae* sp. n.: **C**, male cephalothorax, antero-lateral view; **D**, male pars cephalica and chelicerae, frontal view, showing dorsal ‘head’ region and cheliceral foramen (CF). **E**, Cheliceraeof male *Zephyrarchaea mainae*, lateral view, showing proximal accessory setae (AS) and ectal stridulatory file (SF). **F–G**, Detail of carapace of male *Zephyrarchaea marae*, lateral view, showing granulate cuticle and setose tubercles (sT).<br/>

**Figure 6.**
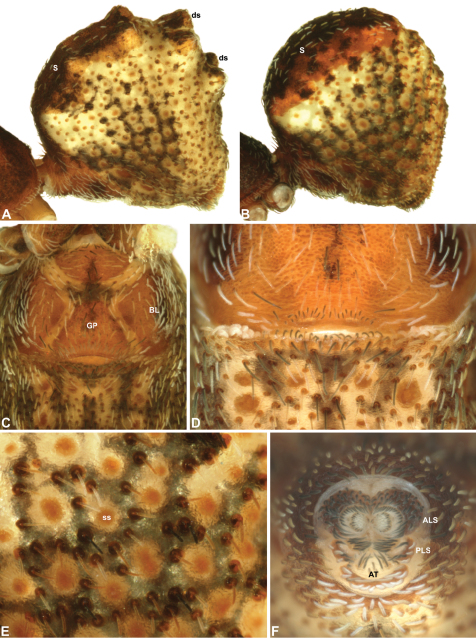
Abdominal morphology of *Zephyrarchaea* species. **A–B**, Male abdomens, dorso-lateral view, showing dorsal scutes (S) and additional dorsal sclerites (ds) on hump-like tubercles: **A**, *Zephyrarchaea mainae* (Platnick); **B**, *Zephyrarchaea marae* sp. n. **C**, Female epigastric region of *Zephyrarchaea mainae*, ventral view, showing setose book lung covers (BL) and median genital plate (GP). **D**, Male epigastric region of *Zephyrarchaea mainae*, ventral view, showing fusion of epigastric sclerites. **E**, Abdominal cuticle of male *Zephyrarchaea marae*, lateral view, showing sclerotic spots (ss) surrounded by short setae. **F**, Spinnerets of female *Zephyrarchaea mainae*, posterior view (ventral side uppermost), showing anterior lateral (ALS) and posterior lateral (PLS) spinnerets anterior to anal tubercle (AT), and the absence of a colulus.

*Colouration*: Body colouration cryptic and relatively uniform across species, usually with only subtle intraspecific variation in abdominal patterning; carapace, sternum and chelicerae tan brown to reddish-brown in males, interspersed with darker regions of granulate cuticle (Figs 5F–G), covered in highly reflective setae; legs tan-brown to darker reddish-brown, with pattern of darker annulations on distal segments; abdomen mottled with beige and variable hues of grey-brown (Fig. 6), with reddish-brown sclerites, scutes and sclerotic spots (Fig. 6); paler beige markings due to reflective, subcuticular guanine crystals; antero-lateral face of abdomen always with large, humeral patch of reflective guanine crystals (Figs 6A–B, 17A–B).

**Figure 7.**
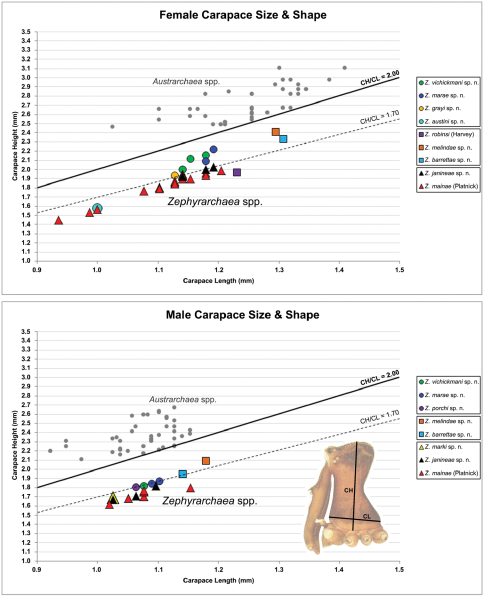
Graphs depicting the relationship between carapace length (CL) and carapace height (CH) for species of *Zephyrarchaea*. Smaller grey dots denote species of *Austrarchaea* (see Rix and Harvey 2011); boxes denote the three lineages of *Zephyrarchaea* from Victoria/South Australia, the Stirling Range and elsewhere in southern Western Australia.<br/>

*Cephalothorax*: Carapace elevated anteriorly (CH/CL ratio usually 1.55–1.75; Fig. 7), with raised, highly modified pars cephalica forming ‘neck’ and bulbous ‘head’ (see Wood 2008; Rix and Harvey 2011) (Figs 5A–D); ‘neck’ with concomitantly long diastema (see Schütt 2002) between cheliceral bases and anterior margin of carapace, fused along entire length with sclerotised cuticle; cheliceral bases emanating from broad, fully-enclosed cheliceral foramen situated at front of ‘head’ (Figs 5B, 5D). Carapace with densely granulate cuticular microstructure, covered in larger setose tubercles arranged in clusters or distinct rows (Figs 5F–G); each tubercle bearing single densely plumose or ciliate seta; setose tubercles largest on ‘neck’ and pars thoracica (Figs 5C, 5F–G). Eight eyes present on anterior margin of ‘head’, in four widely separated diads (Figs 5A–D); AME largest, widely separated, directed antero-laterally on rounded ocular bulge (Fig. 5B); PME situated closely posterior to AME, directed obliquely on postero-lateral side of ocular bulge; lateral eyes contiguous, with shared raised bases, directed ventro-laterally on widest lateral margin of ‘head’ (Figs 5A–D). Sternum longer than wide, covered in setose tubercles; lateral margins separated from dorsal pleural sclerite extending between coxae I–IV. Labium subtriangular, not fused to sternum, directed antero-ventrally at oblique angle to sternum; labrum with pair of divergent projections on anterior surface. Maxillae large, straddling labium and labrum, converging distally; serrula a single row of teeth. Chelicerae very long, spear-like, distally divergent (Figs 5C–E), usually with proximal bulging projection in males (Figs 5A, 5E); both sexes with oval, ectal stridulatory file adjacent to pedipalps (Fig. 5E); males also with tuft (Figs 4B, 5A, 5E, 18B), brush (Figs 14C, 15C) and/or comb (Figs 5C, 16C, 17C) of accessory setae on anterior face of paturon. Chelicerae armed with three rows of peg teeth; anterior (prolateral) row with two peg teeth near tip of fang; posterior (retrolateral) row with single peg tooth near tip of fang; median (prolateral) row with more than 15 peg teeth extending along inner prolateral margin of paturon to near base of fang; median row with approximately eight porrect, comb-like peg teeth adjacent to fang, several larger, flattened, spiniform peg teeth near tip of fang, and additional progressively shorter, spiniform peg teeth along inner paturon; cheliceral retromargin also with four or five true teeth and prominent cheliceral gland mound.

*Legs and female pedipalp*: Legs (longest to shortest) 1–4–2–3, covered with short plumose setae; spines absent; patella I long, greater than one-third length of femur I. Trichobothria present on tibiae and metatarsi of legs; tibiae I–IV each with two trichobothria; metatarsi I–IV each with single trichobothrium. Tarsi shorter than metatarsi, with three claws; tarsi, metatarsi and distal tibiae of legs I–II usually with ventral and pro-ventral rows of moveable, spatulate setae. Female pedipalp with long, porrect trochanter and small tarsal claw; tibia with two dorsal trichobothria.

*Abdomen*: Abdomen arched anteriorly, rounded-subtriangular or spherical in lateral view (Figs 6A–B), sometimes with six large hump-like tubercles on dorsal surface (Figs 6A, 10A–B, 11A–B); cuticle covered with short plumose setae and numerous sclerotic spots (Figs 6C–F). Epigastric region with sclerotised (setose) book lung covers and dorsal and ventral plates surrounding pedicel (Figs 6C–D) (plates fused in males); dorsal pedicel plate with transverse ridges; females with median genital plate and sclerotised lateral sigillae (Fig. 6C); males with broad dorsal scute fused anteriorly to epigastric sclerites, with (Fig. 6A) or usually without (Fig. 6B) additional paired sclerites associated with hump-like tubercles. Six spinnerets, surrounded by thickened cuticle (Fig. 6F); ALS largest, PMS smallest; colulus absent. Posterior pair of divided tracheal spiracles situated closely anterior to spinnerets; males also with epiandrous gland spigots situated closely anterior to epigastric furrow.

*Genitalia*: Female genitalia haplogyne, with sclerotised, subtriangular genital plate anterior to epigastric furrow (Fig. 6C); internally with gonopore leading to large, membranous bursa (Figs 13B–C; see also Harvey 2002a, fig. 2) overlying two separate, radiating clusters of sclerotised, sausage-shaped anterior spermathecae (Figs 13B–D, 15G, 16G). Male pedipalp with complex, expandable spherical or pyriform bulb (Figs 4D, 10E, 16E, 18D), consisting of smooth tegulum, proximal ‘subtegulum’ and associated tegular groove with basal haematodocha (Fig. 4D); distal tegulum with excavate, rimmed cavity surrounding massive, inflatable haematodochal complex, incorporating distal embolus, basal embolic sclerite, two articulating conductor sclerites and three additional tegular sclerites (Figs 4D, 10E, 18D) (see below). Unexpanded pedipalp with folded, curved embolus abutting paired conductor sclerites (Fig. 10E); other tegular sclerites embedded pro-distally (Fig. 10F); pedipalpal expansion and haematodochal inflation (e.g. see Figs 4D, 18D) resulting in significant conformational changes to length and orientation of embolus, and relative position of tegular sclerites.

As noted by Wood (2008) and Rix and Harvey (2011), the homology of the tegular sclerites among archaeid genera remains unclear. Rix and Harvey (2011) used a numbering system for comparing the moveable tegular sclerites among species of *Austrarchaea* from mid-eastern Australia, identifying four separate sclerites (TS 1, 2, 2a and 3) according to their relative position within the unexpanded tegular cavity (see Fig. 4C). These four sclerites can be broadly homologised with the tegular sclerites of species of *Zephyrarchaea*, although at least one sclerite appears to be absent or otherwise highly modified in the latter, with no evidence for an interlocking, differentiated TS 2–2a complex (as in *Austrarchaea*; Fig. 4C cf. Fig. 4D). Tegular sclerite 1 (TS 1) is a prominent and strongly developed process in all species of *Zephyrarchaea* (Figs 4D, 10F, 16F), originating proventrally adjacent to the base of conductor sclerite 1. Two additional tegular sclerites (here labelled collectively TS 2–3) are closely contiguous and not easily distinguished in the unexpanded state, usually embedded pro-distally adjacent to the retro-distal rim of the tegulum. The larger of these two sclerites, presumably homologous to tegular sclerite 3 (TS 3) in species of *Austrarchaea*, has a shorter and broader, more plate-like morphology relative to TS 1, and is usually (but not always) visible as a pointed projection beyond the retro-distal rim of the tegulum (Figs 10E, 16E).

##### Distribution.

Species of *Zephyrarchaea* occur in mesic habitats throughout southern Western Australia, South Australia and Victoria (Fig. 2), usually in coastal (Figs 20C, 22C), sub-coastal or montane (Figs 23C, 24C) temperate heathlands, but also in wet eucalypt forests (Figs 21C, 29C) and temperate rainforests (Figs 26C, 27C). In Victoria they occur along the Great Dividing Range, from Grampians National Park and the Otway Range in south-western Victoria east to the Yarra and Strzelecki Ranges east of Melbourne (Figs 26–29). In South Australia they occur on Kangaroo Island, at a single known locality north of Flinders Chase (Fig. 30). In south-western Western Australia they occur in the southern high rainfall and south-eastern coastal provinces (see Hopper and Gioia 2004; Figs 20–25), from the Wellington and Leeuwin-Naturaliste National Parks (near Bunbury) east to Cape Le Grand National Park, with outlying populations in the Porongurup and Stirling Range National Parks.

##### Composition.

Two described species – *Zephyrarchaea mainae* (Platnick, 1991b) and *Zephyrarchaea robinsi* (Harvey, 2002a) – and the nine new species from southern Australia: *Zephyrarchaea austini* sp. n., *Zephyrarchaea barrettae* sp. n., *Zephyrarchaea grayi* sp. n., *Zephyrarchaea janineae* sp. n., *Zephyrarchaea marae* sp. n., *Zephyrarchaea marki* sp. n., *Zephyrarchaea melindae* sp. n., *Zephyrarchaea porchi* sp. n. and *Zephyrarchaea vichickmani* sp. n. The previously described species *Archaea hickmani* Butler, 1929 is here recognised as a nomen dubium.

##### Remarks.

The genus *Zephyrarchaea* forms a monophyletic and highly divergent clade sister to all other Archaeidae from mid-eastern and north-eastern Australia (see Rix and Harvey 2011, 2012; Fig. 3). Three main lineages have been recognised within the genus, for species from south-eastern Australia (South Australia and Victoria), from the Stirling Range National Park and from elsewhere in south-western Western Australia (see Rix and Harvey 2012; Fig. 3). The genus is not known to occur north or east of the Australian Alps, which may be a vicariant biogeographic barrier between populations of *Zephyrarchaea* and *Austrarchaea*.

##### Key to the Australian species of 

***Zephyrarchaea***

Note that males of *Zephyrarchaea austini* sp. n., *Zephyrarchaea grayi* sp. n. and *Zephyrarchaea robinsi* are unknown; females of *Zephyrarchaea marki* sp. n. and *Zephyrarchaea porchi* sp. n. are unknown.

**Table:** 

1	Males	2
–	Females	9
2	Abdomen with six pronounced dorsal hump-like tubercles (HT 1–6), in three pairs, HT 3–6 each with small dorsal sclerite posterior to dorsal scute (Figs 6A, 10B, 11B)	3
–	Abdomen spherical, or nearly so, without pronounced hump-like tubercles and without additional sclerites posterior to dorsal scute (Figs 1B, 1D, 6B, 12A, 14B, 15B, 16B, 17B, 18A)	4
3	Tegular sclerite 1 (TS 1) with flattened, rounded apex (Figs 10D–F)	*Zephyrarchaea mainae* (Platnick, 1991b)
–	Tegular sclerite 1 (TS 1) with more tapered, spiniform apex (Figs 11D–F)	*Zephyrarchaea janineae* sp. n.
4	Chelicerae with proximal bulging projection bearing tuft or brush of accessory setae (Figs 12B, 14C, 15C, 18B)	5
–	Chelicerae without tuft of accessory setae on proximal bulging projection (Figs 16C, 17C)	8
5	Tegular sclerites 2–3 (TS 2–3) not projecting beyond retro-distal margin of tegulum (Figs 14D–E, 15D–E)	6
–	Tegular sclerites 2–3 (TS 2–3) projecting well beyond retro-distal margin of tegulum (Figs 12C–D, 18C–D)	7
6	Anterior margin of diastema adjacent to ‘neck’ almost straight, only slightly concave in lateral view (Fig. 14B)	*Zephyrarchaea melindae* sp. n.
–	Anterior margin of diastema adjacent to ‘neck’ curved, strongly concave in lateral view (Fig. 15B)	*Zephyrarchaea barrettae* sp. n.
7	Proximal bulging projection on chelicerae strongly protuberant (Fig. 12B); dorsal scute on abdomen extending posteriorly (in lateral view) to roughly in-line with epigastric furrow (Fig. 12A)	*Zephyrarchaea marki* sp. n.
–	Proximal bulging projection on chelicerae indistinct, only slightly protuberant (Fig. 18B); dorsal scute on abdomen larger, extending posteriorly (in lateral view) to beyond line of epigastric furrow (Fig. 18A)	*Zephyrarchaea porchi* sp. n.
8	Conductor sclerite 2 (C2) relatively slender, with sinuous, S-shaped proximal portion (Figs 17D–E); anterior margin of diastema adjacent to ‘neck’ slightly convex in lateral view (Fig. 17B)	*Zephyrarchaea marae* sp. n.
–	Conductor sclerite 2 (C2) without sinuous, S-shaped proximal portion (Figs 16D–E); anterior margin of diastema adjacent to ‘neck’ slightly concave in lateral view (Fig. 16B)	*Zephyrarchaea vichickmani* sp. n.
9	Abdomen with six pronounced dorsal hump-like tubercles (HT 1–6), in three pairs (Figs 1C, 1E–F, 10A, 11A)	10
–	Abdomen spherical, or nearly so, without pronounced dorsal hump-like tubercles (Figs 1A, 13A, 14A, 15A, 16A, 17A, 19A, 19C)	11
10	‘Head’ not strongly elevated dorsally, post-ocular ratio < 0.25 (Fig. 9E); highest point of pars cephalica (HPC) near posterior third of ‘head’, ratio of HPC to post-ocular length ≤ 0.66 (Fig. 9E)	*Zephyrarchaea mainae* (Platnick, 1991b)*
–	‘Head’ more strongly elevated dorsally, post-ocular ratio ≥ 0.25 (Fig. 9F); highest point of pars cephalica (HPC) approaching posterior quarter of ‘head’, ratio of HPC to post-ocular length > 0.66 (Fig. 9F)	*Zephyrarchaea janineae* sp. n.*
11	Carapace with strongly concave post-ocular depression in lateral view (Figs 9A–D)	12
–	Carapace with shallow post-ocular depression in lateral view (Figs 9G–I)	14
12	Body size small, carapace length < 1.10 (Fig. 7); carapace relatively short, CH/CL ratio < 1.70 (Figs 7, 19C)	*Zephyrarchaea austini* sp. n.
–	Body size larger, carapace length > 1.10 (Fig. 7); carapace taller, CH/CL ratio ≥ 1.70 (Figs 7, 19A)	13
13	‘Head’ not strongly elevated dorsally, post-ocular ratio < 0.25 (Fig. 9C); highest point of pars cephalica (HPC) approaching middle of ‘head’, ratio of HPC to post-ocular length < 0.60 (Fig. 9C)	*Zephyrarchaea grayi* sp. n.
–	‘Head’ more strongly elevated dorsally, post-ocular ratio ≥ 0.25 (Figs 9A–B); highest point of pars cephalica (HPC) approaching posterior third of ‘head’, ratio of HPC to post-ocular length > 0.60 (Figs 9A–B)	*Zephyrarchaea marae* sp. n./*Zephyrarchaea vichickmani* sp. n.**
14	Carapace relatively short, CH/CL ratio < 1.70 (Figs 7, 13A)	*Zephyrarchaea robinsi* (Harvey, 2002a)
–	Carapace taller, CH/CL ratio > 1.70 (Figs 7, 14A, 15A)	15
15	Anterior margin of diastema adjacent to ‘neck’ straight, almost perpendicular in lateral view (Fig. 14A)	*Zephyrarchaea melindae* sp. n.
–	Anterior margin of diastema adjacent to ‘neck’ slightly curved, concave in lateral view (Fig. 15A)	*Zephyrarchaea barrettae* sp. n.

* Females of *Zephyrarchaea mainae* and *Zephyrarchaea janineae* sp. n. are very similar morphologically, with only subtle morphometric differences in the shape of the carapace; male specimens or nucleotide sequences are recommended to accurately identify these closely related species.

** Females of *Zephyrarchaea vichickmani* sp. n. and *Zephyrarchaea marae* sp. n. are essentially indistinguishable morphologically, with male specimens or nucleotide sequences required to identify these closely related sister-species.

### The Western Australian fauna

#### Zephyrarchaea mainae

(Platnick, 1991b) comb. n.

http://species-id.net/wiki/Zephyrarchaea_mainae

Figs 1E–F 5A–B, 5E 6A, 6C–D, 6F 8G 9E 10 20

Austrarchaea mainae Platnick, 1991b: 259, figs 1–7.

Albany Assassin Spider

##### Type material.

Holotype male. Torndirrup National Park (probably near end of Eclipse Island Road), Western Australia, Australia, pitfall trap, 1–6.VI.1983, P. Dyer, J. Lyon (WAM T17683).

Paratypes. Allotype female, same data as holotype except 3–8.X.1983 (WAM T17684).

##### Other material examined.

**AUSTRALIA: *Western Australia*:** **Torndirrup National Park:** same data as holotype except 2–9.XI.1983, 1 juvenile (WAM T17682); next to carpark at end of Salmon Hole Road, 35°06'07"S, 117°58'03"E, sifting elevated leaf litter under *Agonis*, 14.III.2008, M. Rix, M. Harvey, 1♀, 2 juveniles (WAM T89566^DNA: TO-1-J/TO-2-J^); same data except 30 April 2008, 1♀ (AMNH); same data, 1♀ (QMB S91206); same data, 1 juvenile (WAM T89567^DNA: TO-171-J^); next to carpark at base of Isthmus Hill, 35°05'55"S, 117°58'02"E, sifting elevated leaf litter in coastal *Agonis* and eucalypt grove, 14.III.2008, M. Rix, M. Harvey, 2 juveniles (WAM T89568^DNA: TO-3-J/TO-4-J^). **Bremer Bay:** Wellstead Road, S. of Bremer Bay, near Yate Place, 34°24'10"S, 119°22'42"E, sifting elevated leaf litter in *Agonis* grove, 19.VI.2010, M. Rix, J.D. Roberts, 1♂, 1♀ (WAM T118984); same data except 21.IV.2009, M. Rix, 1♂, 1 juvenile (WAM T97463^DNA: BB-147-J^); same data except 2.V.2008, M. Rix, M. Harvey, J. Newell, 1♀, 1 juvenile (WAM T89563^DNA: BB-34-F/BB-35-J^). **Denmark Region:** Gilge Road, 15 km SE. of Denmark, 35°03'15"S, 117°28'49"E, sifting elevated leaf litter in remnant Karri grove with *Agonis*, 16.III.2008, M. Rix, M. Harvey, 1♀, 1 juvenile (WAM T89577^DNA: GI-14-F/GI-15-J^); Mount Hallowell, 7 km SW. of Denmark, 35°00'34"S, 117°18'04"E, beating grass clumps in Karri forest, 6.XI.2007, M. Moir, D. Jolly, 1 juvenile (WAM T78901); Mount Hallowell, 7 km SW. of Denmark, 35°00'38"S, 117°17'57"E, beating grass clump in Karri forest, 19.III.2008, M. Rix, M. Harvey, 1 juvenile (WAM T89590^DNA: MH-30-J^). **Gull Rock National Park:** end of Ledge Point Road, near carpark, 35°00'51"S, 118°00'23"E, sifting elevated leaf litter under *Agonis*, 17.III.2008, M. Rix, M. Harvey, 1♀, 1 juvenile (WAM T89578^DNA: GR-16-F/GR-17-J^). **Mutton Bird Point:** end of Mutton Bird Road, 35°02'53"S, 117°41'42"E, sifting elevated leaf litter under *Agonis*, 19.VI.2010, M. Rix, J.D. & B. Roberts, 1♂, 2♀, 2 juveniles (WAM T118983); end of Mutton Bird Road, 35°02'52"S, 117°41'42"E, sifting elevated leaf litter under *Agonis*, 18.III.2008, M. Rix, M. Harvey, 1♂, 3 juveniles (WAM T89582^DNA: MB-21-J^); end of Mutton Bird Road, 35°02'40"S, 117°41'50"E, sifting elevated leaf litter under *Agonis*, 18.III.2008, M. Rix, M. Harvey, 1♀ (WAM T89581^DNA: MB-20-F^); end of Mutton Bird Road, 35°02'54"S, 117°42'07"E, sifting elevated leaf litter under *Agonis*, 18.III.2008, M. Rix, M. Harvey, 1♀ (WAM T89584^DNA: MB-23-F^); end of Mutton Bird Road, 35°02'42"S, 117°41'36"E, sifting elevated leaf litter under *Agonis*, 18.III.2008, M. Rix, M. Harvey, 1 juvenile (WAM T89579); end of Mutton Bird Road, 35°02'43"S, 117°41'43"E, sifting elevated leaf litter under *Agonis*, 18.III.2008, M. Rix, M. Harvey, 1 juvenile (WAM T89580); end of Mutton Bird Road, 35°02'58"S, 117°41'56"E, sifting elevated leaf litter under *Agonis*, 18.III.2008, M. Rix, M. Harvey, 1 juvenile (WAM T89583). **Porongurup National Park:** west of Waddy's Hut, 34°40'54"S, 117°50'48"E, sifting elevated leaf litter in unburnt Karri forest, 306 m, 20.IV.2009, M. Rix, 1♀, 1 juvenile (WAM T97465^DNA: PO-166-F/PO-167-J^); same data except 6 March 2010, M. Rix, L. Lopardo, 1♀ (WAM T114028^DNA: PO-168-F^). **Torndirrup Peninsula:** Albany Wind Farm, 35°03'53"S, 117°47'35"E, beating vegetation in coastal heathland, 26.IX.2007, M. Harvey, R. Ott, 3 juveniles (WAM T89557); Albany Wind Farm, off Bibbulmun Track boardwalk, 35°03'57"S, 117°47'43"E, sifting elevated leaf litter under *Banksia praemorsa*, 12.III.2007, M. Rix, M. Harvey, M. Moir, 1 juvenile (WAM T81097); Albany Wind Farm, NW. of Wind Turbine No. 2, 35°03'43"S, 117°47'46"E, sifting elevated leaf litter under *Agonis*, 15.III.2008, M. Rix, M. Harvey, 1 juvenile (WAM T89575^DNA: WF-12-J^); Albany Wind Farm, NW. of Wind Turbine No. 3, 35°03'44"S, 117°47'38"E, sifting elevated leaf litter under *Agonis*, 15.III.2008, M. Rix, M. Harvey, 1 juvenile (WAM T89576^DNA: WF-13-J^); Albany Wind Farm, SW. of Wind Turbine No. 5, 35°03'35"S, 117°47'22"E, sifting elevated leaf litter under *Agonis*, 15.III.2008, M. Rix, M. Harvey, 1♀ (WAM T89574^DNA: WF-11-F^); along Bibbulmun Track E. of Albany Wind Farm, 35°04'05"S, 117°48'01"E, sifting elevated leaf litter under *Agonis*, 27.V.2011, M. Rix, M. Harvey, G. Binford, 1♂ (WAM T118982); along Bibbulmun Track E. of Albany Wind Farm, 35°04'05"S, 117°48'07"E, sifting elevated leaf litter under *Agonis*, 18.III.2008, M. Rix, M. Harvey, 1♀ (WAM T89585^DNA: WF-24-F^); along Bibbulmun Track E. of Albany Wind Farm, 35°04'10"S, 117°48'26"E, sifting elevated leaf litter under *Agonis*, 18.III.2008, M. Rix, M. Harvey, 1 juvenile (WAM T89588); along Bibbulmun Track E. of Albany Wind Farm, 35°04'08"S, 117°48'18"E, sifting elevated leaf litter under *Agonis*, 18.III.2008, M. Rix, M. Harvey, 1 juvenile (WAM T89587^DNA: WF-26-J^); along Bibbulmun Track E. of Albany Wind Farm, 35°04'06"S, 117°48'09"E, sifting elevated leaf litter under *Agonis*, 18.III.2008, M. Rix, M. Harvey, 1 juvenile (WAM T89586^DNA: WF-25-J^); Cuthbert, W. of Roberts Road, 35°01'59"S, 117°47'47"E, sifting elevated leaf litter under *Agonis*, 18.III.2008, M. Rix, M. Harvey, 1♀, 2 juveniles (WAM T89589^DNA: WF-28-J/WF-29-J^); end of Prescott Vale Road, W. of Albany Wind Farm, 35°02'57"S, 117°45'30"E, sifting elevated leaf litter under *Agonis*, 15.III.2008, M. Rix, M. Harvey, 1♂, 3 juveniles (WAM T89569^DNA: WF-5-J/WF-6-J^); end of Prescott Vale Road, W. of Albany Wind Farm, 35°03'10"S, 117°46'11"E, sifting elevated leaf litter under *Agonis*, 15.III.2008, M. Rix, M. Harvey, 1 juvenile (WAM T89573^DNA: WF-10-J^); end of Prescott Vale Road, W. of Albany Wind Farm, 35°03'10"S, 117°45'59"E, sifting elevated leaf litter under *Agonis*, 15.III.2008, M. Rix, M. Harvey, 1♀ (WAM T89572^DNA: WF-9-F^); end of Prescott Vale Road, W. of Albany Wind Farm, 35°03'02"S, 117°45'28"E, sifting elevated leaf litter under *Agonis*, 15.III.2008, M. Rix, M. Harvey, 1♀ (WAM T89570); end of Prescott Vale Road, W. of Albany Wind Farm, 35°03'10"S, 117°45'43"E, sifting elevated leaf litter under *Agonis*, 15.III.2008, M. Rix, M. Harvey, 1♀ (WAM T89571^DNA: WF-8-F^). **Walpole-Nornalup National Park:** Anderson Road, 34°59'43"S, 116°52'14"E, sifting elevated leaf litter under curly grass in Marri and *Agonis* forest, 3.V.2008, M. Rix, M. Harvey, 2 juveniles (WAM T89564^DNA: WA-36-J/WA-37-J^); same data except 24.I.2010, M. Rix, J. Wojcieszek, 1♂, 2♀, 1 juvenile (WAM T114037^DNA: WA-162-F^). **William Bay National Park:** near Elephant Rock carpark, 35°01'21"S, 117°14'12"E, sifting elevated leaf litter under *Agonis*, 26.VI.2010, M. Rix, 2♀ (WAM T114029^DNA: WB-169-F^); same data except 29.IV.2008, M. Rix, M. Harvey, 1 juvenile (WAM T89591^DNA: WB-31-J^); same data except 7.III.2010, M. Rix, L. Lopardo, 2 juveniles (WAM T114030^DNA: WB-170-J^).

##### Diagnosis.

*Zephyrarchaea mainae* can be distinguished from other known congeners except *Zephyrarchaea janineae* sp. n. by the presence of six dorsal, hump-like tubercles on the abdomen (Figs 1E–F, 6A, 10A–B); and from *Zephyrarchaea janineae* sp. n. by the more flattened, rounded apex of tegular sclerite 1 (TS 1) (Figs 10D–F).

This species can also be distinguished from other genotyped taxa (see Fig. 3) by the following two unique nucleotide substitutions for COI and COII (n = 35): A(282), T(1173).

##### Description.

**Figure 8.**
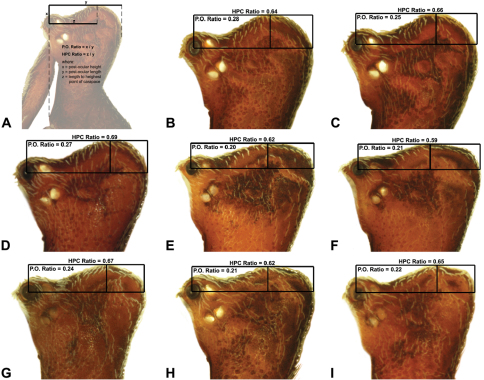
Lateral ‘head’ profiles of males of species of *Zephyrarchaea*, showing variation in carapace shape as quantified by the post-ocular ratio (P.O. Ratio) and ratio of highest point of carapace relative to post-ocular length (HPC Ratio): **A**, holotype *Zephyrarchaea marae* sp. n., showing the derivation of morphometric ratios; **B**, holotype *Zephyrarchaea vichickmani* sp. n.; **C**, holotype *Zephyrarchaea marae* sp. n.; **D**, holotype *Zephyrarchaea porchi* sp. n.; **E**, holotype *Zephyrarchaea melindae* sp. n.; **F**, holotype *Zephyrarchaea barrettae* sp. n.; **G**, holotype *Zephyrarchaea mainae* (Platnick, 1991b); **H**, holotype *Zephyrarchaea marki* sp. n.; **I**, holotype *Zephyrarchaea janineae* sp. n.<br/>

*Holotype male*: Total length 3.44; leg I femur 1.81; F1/CL ratio 1.72. Cephalothorax dark reddish-brown; legs tan brown with darker annulations; abdomen mottled grey-brown and beige, with reddish-brown dorsal scute and sclerites (Fig. 10B). Carapace short (CH/CL ratio 1.60); 1.05 long, 1.68 high, 1.03 wide; ‘neck’ 0.60 wide; highest point of pars cephalica (HPC) near posterior third of ‘head’ (ratio of HPC to post-ocular length 0.67), carapace with pronounced concave depression anterior to HPC; ‘head’ not strongly elevated dorsally (post-ocular ratio 0.24) (Fig. 8G). Chelicerae with proximal tuft and additional comb of accessory setae on anterior face of paturon (Figs 5A, 5E, 10C). Abdomen 1.69 long, 1.18 wide; with three pairs of dorsal hump-like tubercles (HT 1–6); dorsal scute fused anteriorly to epigastric sclerites, extending posteriorly to first pair of hump-like tubercles; HT 3–6 each covered by separate dorsal sclerites. Pedipalp fully expanded (see Platnick 1991b, figs 4–6). Unexpanded pedipalp (of WAM T89569) (Figs 10D–F) pyriform, with broad, distally curved embolus supported by conductor sclerites 1–2; tegular sclerite 1 (TS 1) porrect, slightly curved in prolateral view, with flattened, rounded apex; TS 2–3 projecting beyond retro-distal rim of tegulum.

**Figure 9.**
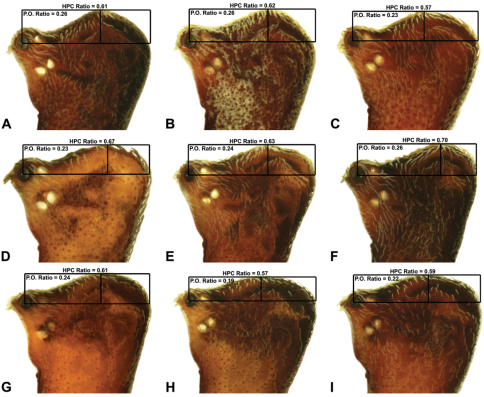
Lateral ‘head’ profiles of females of species of *Zephyrarchaea*, showing variation in carapace shape as quantified by the post-ocular ratio (P.O. Ratio) and ratio of highest point of carapace relative to post-ocular length (HPC Ratio) (see Fig. 8): **A**, allotype *Zephyrarchaea vichickmani* sp. n.; **B**, allotype *Zephyrarchaea marae* sp. n.; **C**, holotype *Zephyrarchaea grayi* sp. n.; **D**, holotype *Zephyrarchaea austini* sp. n.; **E**, *Zephyrarchaea mainae* (Platnick, 1991b); **F**, allotype *Zephyrarchaea janineae* sp. n.; **G**, holotype *Zephyrarchaea robinsi* (Harvey, 2002a); **H**, allotype *Zephyrarchaea melindae* sp. n.; **I**, allotype *Zephyrarchaea barrettae* sp. n.

**Figure 10.**
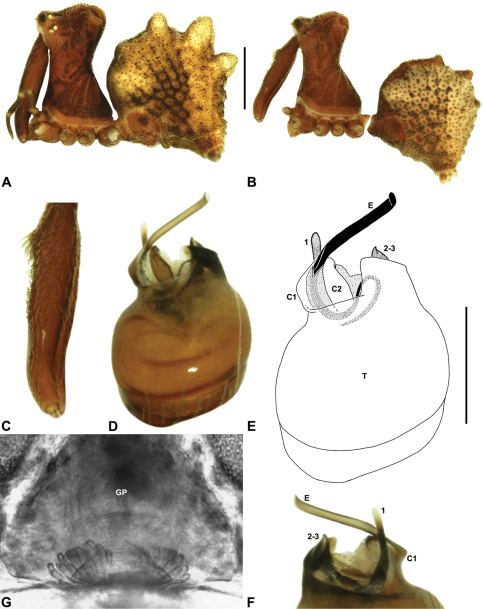
*Zephyrarchaea mainae* (Platnick, 1991b). **A–B**, Cephalothorax and abdomen, lateral view: **A**, female (WAM T118983) from Mutton Bird Point, Western Australia; **B**, holotype male (WAM T17683) from Torndirrup National Park, Western Australia. **C**, Holotype male chelicerae, lateral view, showing accessory setae. **D–F**, Male (WAM T89569) pedipalp: **D–E**, bulb, retrolateral view; **F**, detail of distal tegular sclerites, prolateral view. **G**, Female (WAM T118983) internal genitalia, dorsal view. C1–2 = conductor sclerites 1–2; E = embolus; GP = genital plate; T = tegulum; (TS)1–3 = tegular sclerites 1–3. Scale bars: A–B = 1.0 mm; E = 0.2 mm.<br/>

*Female* (WAM T118983): Total length 3.31; leg I femur 1.97; F1/CL ratio 1.73. Cephalothorax dark reddish-brown; legs tan brown with darker annulations; abdomen mottled grey-brown and beige (Fig. 10A). Carapace short (CH/CL ratio 1.71); 1.14 long, 1.95 high, 1.10 wide; ‘neck’ 0.68 wide; highest point of pars cephalica (HPC) near posterior third of ‘head’ (ratio of HPC to post-ocular length 0.63), carapace with pronounced concave depression anterior to HPC; ‘head’ not strongly elevated dorsally (post-ocular ratio 0.24) (Fig. 9E). Chelicerae without accessory setae on anterior face of paturon. Abdomen 2.00 long, 1.36 wide; with three pairs of dorsal hump-like tubercles (HT 1–6). Internal genitalia (Fig. 10G) with cluster of ≤ 15 sausage-shaped spermathecae either side of gonopore, clusters widely separated along midline of genital plate.

*Variation*: Males (n = 8): total length 2.82–3.44; carapace length 1.02–1.15; carapace height 1.62–1.80; CH/CL ratio 1.56–1.64. Females (n = 23): total length 2.77–3.82; carapace length 0.94–1.21; carapace height 1.45–1.99; CH/CL ratio 1.55–1.71. Specimens of *Zephyrarchaea mainae* from the eastern-most Bremer Bay population have significantly smaller hump-like tubercles on the abdomen compared to populations further west (see Fig. 1F cf. Figs 1E, 10A), and males from this Bremer Bay population also have a more distally paddle-shaped TS 1 morphology similar to *Zephyrarchaea marki* sp. n. from Cape Le Grand National Park. Rix and Harvey (2012) showed that the Bremer Bay population of *Zephyrarchaea mainae* is genetically distinct (Fig. 3), with no evidence of recent gene flow, indicating possible incipient speciation.

##### Distribution and habitat.

*Zephyrarchaea mainae* is known from the greater Albany region of southern Western Australia, from Walpole-Nornalup National Park (near Walpole) east to Bremer Bay and north to the Porongurup National Park, with a range centred on the Torndirrup Peninsula south of Albany (Fig. 20). Specimens have been collected by beating and sifting sedges (*Lepidosperma* sp.), curly grass (*Empodisma gracillimum*) and low shrubs in dense coastal or near-coastal groves of Peppermint (*Agonis* sp.), with several outlying populations also known from wet Karri (*Eucalyptus diversicolor*) forest.

##### Conservation status.

Thisspecies is listed as **threatened** under the*Western Australian Wildlife Conservation Act 1950*. It is a short-range endemic taxon (Harvey 2002b), with known populations threatened by fire, dieback disease (affecting coastal heathland vegetation), land-clearing and climate change.

#### Zephyrarchaea janineae

Rix & Harvey sp. n.

urn:lsid:zoobank.org:act:3E20D50E-50A2-4D59-A2CD-565E05D4554D

http://species-id.net/wiki/Zephyrarchaea_janineae

Figs 1C 8I 9F 11 21

Austrarchaea sp. Main, 1995: 153.

Karri Forest Assassin Spider

##### Type material.

Holotype male: Karri Valley, ‘Karri Valley Hideaway Cottages’, off Hopgarden Road, west of Pemberton, Western Australia, Australia, 34°24'59"S, 115°50'52"E, sifting elevated leaf litter in wet Marri and *Agonis* forest, 26–28.VIII.2006, M. Rix, J. Wojcieszek (WAM T89559).

Paratypes: Allotype female, same data as holotype (WAM T118981).

##### Other material examined.

**AUSTRALIA: *Western Australia*:** **Karri Valley:** ‘Karri Valley Hideaway Cottages’, off Hopgarden Road, west of Pemberton, 34°24'57"S, 115°50'50"E, sifting elevated leaf litter in wet Marri and *Agonis* forest, 3.V.2008, M. Rix, M. Harvey, 2 juveniles (WAM T89565^DNA: KV-38-J/KV-39-J^). **Greater Hawke National Park:** Gloucester Road, ~360 m off Pemberton-Northcliffe Road, 34°21'01"S, 116°01'14"E, sifting leaf litter and beating grass-trees in very wet area, 16.X.2009, D. & S. Harms, 1 juvenile (WAM T114035^DNA: GL-160-J^). **Dombakup State Forest:** Marri Road, 15.I.1979, M. Gray, 4 juveniles (AMS KS15242). **Leeuwin-Naturaliste National Park:** near Cape Leeuwin, 34°22'00"S, 115°09'16"E, sifting elevated leaf litter in coastal *Agonis* forest, 15.VII.2009, M. Rix, 2♂ (WAM T94476); same data except 16.IV.2009, 1♀, 5 juveniles (WAM T97464^DNA: CL-163-J/CL-164-J/CL-165-J^); Sugarloaf Road, near Cape Naturaliste, 33°33'31"S, 115°01'18"E, sifting elevated leaf litter under *Agonis*, 30.III.2008, M. Rix, 1 juvenile (WAM T89561^DNA: CN-32-J^); Sugarloaf Road, near Cape Naturaliste, 33°33'29"S, 115°01'25"E, sifting elevated leaf litter under *Agonis*, 25.IV.2008, M. Rix, 2 juveniles (WAM T89562^DNA: CN-33-J^). **Treen Brook State Forest:** side road fire track off Vasse Highway, 34°26'45"S, 115°59'00"E, sifting leaf litter and teasing low shrubs, 14.X.2009, D. & S. Harms, 1 juvenile (WAM T114036^DNA: TB-161-J^); 8 km W. of Pemberton, 13.II.1979, M. Gray, 2 juveniles (AMS KS15341). **Wellington National Park:** Lennard Drive, near turnoff to Rapids Picnic Ground, 33°23’59”S, 115°57’52”E, sifting elevated leaf litter, dense Jarrah forest with *Agonis* on slope leading to Collie River, 141 m, 18.IX.2010, S. & D. Harms, 1♀ (WAM T112584^DNA: CO-158-F^); same data except 25.IX.2010, M. Rix, J. Wojcieszek, 1 juvenile (WAM T114034^DNA: CO-159-J^).

##### Etymology.

The specific epithet is a patronym in honour of Dr Janine Wojcieszek, for helping to discover the first live specimens of this species in 2006, and therefore catalysing the Western Australian Museum’s ‘archaeid project’ in the half decade since 2007.

##### Diagnosis.

**Figure 11.**
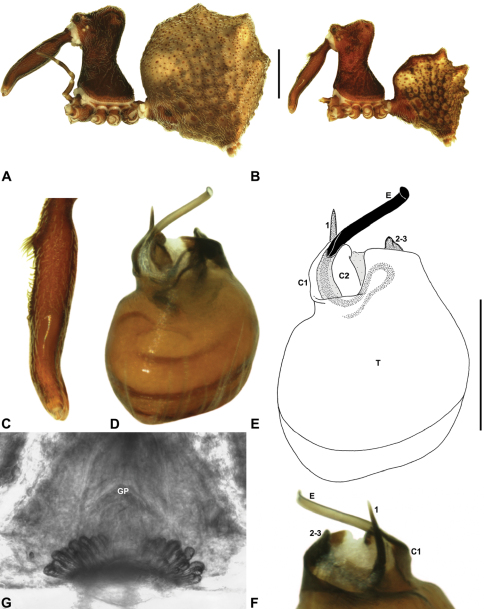
*Zephyrarchaea janineae* sp. n. **A–B**, Cephalothorax and abdomen, lateral view: **A**, allotype female (WAM T118981) from Karri Valley, Western Australia; **B**, holotype male (WAM T89559) from Karri Valley, Western Australia. **C**, Holotype male chelicerae, lateral view, showing accessory setae. **D–F**, Holotype male pedipalp: **D–E**, bulb, retrolateral view; **F**, detail of distal tegular sclerites, prolateral view. **G**, Allotype female internal genitalia, dorsal view. C1–2 = conductor sclerites 1–2; E = embolus; GP = genital plate; T = tegulum; (TS)1–3 = tegular sclerites 1–3. Scale bars: A–B = 1.0 mm; E = 0.2 mm.

*Zephyrarchaea janineae* can be distinguished from other known congeners except *Zephyrarchaea mainae* by the presence of six dorsal, hump-like tubercles on the abdomen (Figs 1C, 11A–B); and from *Zephyrarchaea mainae* by the more tapered, spiniform apex of tegular sclerite 1 (TS 1) (Figs 11D–F).

This species can also be distinguished from other genotyped taxa (see Fig. 3) by the following three unique nucleotide substitutions for COI (n = 11): G(87), G(132), T(609).

##### Description.

*Holotype male*: Total length 2.92; leg I femur 1.87; F1/CL ratio 1.76. Cephalothorax dark reddish-brown; legs tan brown with darker annulations; abdomen mottled grey-brown and beige, with reddish-brown dorsal scute and sclerites (Fig. 11B). Carapace short (CH/CL ratio 1.60); 1.06 long, 1.71 high, 1.01 wide; ‘neck’ 0.59 wide; highest point of pars cephalica (HPC) near posterior third of ‘head’ (ratio of HPC to post-ocular length 0.65), carapace with pronounced concave depression anterior to HPC; ‘head’ not strongly elevated dorsally (post-ocular ratio 0.22) (Fig. 8I). Chelicerae with proximal tuft and additional comb of accessory setae on anterior face of paturon (Fig. 11C). Abdomen 1.54 long, 1.08 wide; with three pairs of dorsal hump-like tubercles (HT 1–6); dorsal scute fused anteriorly to epigastric sclerites, extending posteriorly to first pair of hump-like tubercles; HT 3–6 each covered by separate dorsal sclerites. Unexpanded pedipalp (Figs 11D–F) pyriform, with broad, distally curved embolus supported by conductor sclerites 1–2; tegular sclerite 1 (TS 1) porrect, slightly curved in prolateral view, with tapered, spiniform apex; TS 2–3 projecting beyond retro-distal rim of tegulum.

*Allotype female*: Total length 4.10; leg I femur 1.97; F1/CL ratio 1.66. Cephalothorax dark reddish-brown; legs tan brown with darker annulations; abdomen mottled grey-brown and beige (Fig. 11A). Carapace short (CH/CL ratio 1.70); 1.19 long, 2.03 high, 1.15 wide; ‘neck’ 0.69 wide; highest point of pars cephalica (HPC) approaching posterior quarter of ‘head’ (ratio of HPC to post-ocular length 0.70), carapace with pronounced concave depression anterior to HPC; ‘head’ moderately elevated dorsally (post-ocular ratio 0.26) (Fig. 9F). Chelicerae without accessory setae on anterior face of paturon. Abdomen 2.62 long, 2.15 wide; with three pairs of dorsal hump-like tubercles (HT 1–6). Internal genitalia (Fig. 11G) with cluster of ≤ 15 sausage-shaped spermathecae either side of gonopore, clusters widely separated along midline of genital plate.

*Variation*: Males (n = 3): total length 2.92–3.15; carapace length 1.03–1.10; carapace height 1.67–1.82; CH/CL ratio 1.60–1.66. Females (n = 3): total length 3.03–4.10; carapace length 1.14–1.19; carapace height 1.92–2.03; CH/CL ratio 1.69–1.70.

##### Distribution and habitat.

*Zephyrarchaea janineae* is known from the high rainfall province (see Hopper and Gioia 2004) of southern Western Australia, from the Leeuwin-Naturaliste and Wellington National Parks (near Bunbury) east to Pemberton (Fig. 21). It is the dominant assassin spider of the south-western Karri (*Eucalyptus diversicolor*) forest and surrounding areas, and has been collected by beating and sifting elevated leaf litter in wet forested habitats and in coastal groves of Peppermint (*Agonis* sp.). Six juvenile specimens first collected by M. Gray in 1979 in the Treen Brook and Dombakup State Forests near Pemberton (see Main 1995) almost certainly belong to this species.

##### Conservation status.

This specieshas a relatively widespread distribution in several National Parks and State Forests, and is not considered to be of conservation concern.

#### Zephyrarchaea marki

Rix & Harvey sp. n.

urn:lsid:zoobank.org:act:8EF9792F-4867-40B9-A409-2318A03E33E8

http://species-id.net/wiki/Zephyrarchaea_marki

Figs 1D 4B 8H 12 22

Cape Le Grand Assassin Spider

##### Type material.

Holotype male: Cape Le Grand National Park, Thistle Cove, Western Australia, Australia, 33°59'55"S, 122°11'59"E, sifting elevated leaf litter in *Banksia speciosa* thicket behind beach, 5.VI.2010, M. Rix (WAM T118985).

Paratypes: 1 male and 1 juvenile, same data as holotype (WAM T114033^DNA: CLG-146-J^).

##### Other material examined.

**AUSTRALIA: *Western Australia*:** **Cape Le Grand National Park:** Thistle Cove, 33°59'54"S, 122°12'01"E, sifting elevated leaf litter, *Banksia speciosa* thicket behind beach, 28.VII.2009, M. Rix, M. Wojcieszek, 3 juveniles (WAM T94477^DNA: CLG-144-J/CLG-145-J^).

##### Etymology.

The specific epithet is a patronym in honour of Mark Wojcieszek, for helping to discover the first specimens of this species at Cape Le Grand National Park in 2009.

##### Diagnosis.

**Figure 12.**
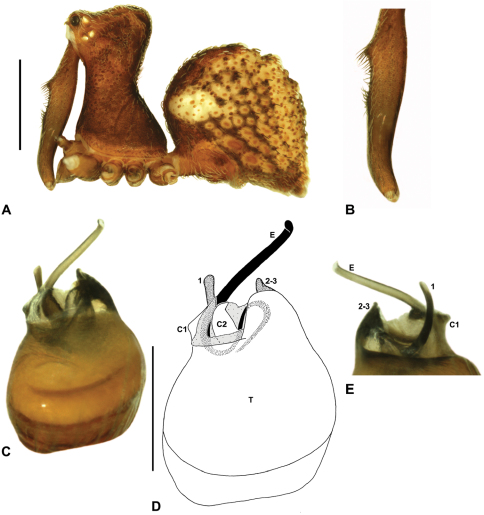
*Zephyrarchaea marki* sp. n. **A–E**, Holotype male (WAM T118985) from Thistle Cove, Cape Le Grand National Park, Western Australia: **A**, cephalothorax and abdomen, lateral view; **B**, chelicerae, lateral view, showing accessory setae; **C–D**, pedipalpal bulb, retrolateral view; **E**, detail of distal tegular sclerites, prolateral view. C1–2 = conductor sclerites 1–2; E = embolus; T = tegulum; (TS)1–3 = tegular sclerites 1–3. Scale bars: A = 1.0 mm; D = 0.2 mm.<br/>

*Zephyrarchaea marki* can be distinguished from *Zephyrarchaea janineae* and *Zephyrarchaea mainae* by the absence of dorsal hump-like tubercles on the abdomen (Fig. 12A); from *Zephyrarchaea marae* sp. n. and *Zephyrarchaea vichickmani* sp. n. by the presence of a proximal tuft of accessory setae on the male chelicerae (Fig. 12B); from *Zephyrarchaea barrettae* sp. n. and *Zephyrarchaea melindae* sp. n. by the shape of tegular sclerites 2–3, which project well beyond the retro-distal rim of the tegulum (Figs 12C–D); and from *Zephyrarchaea porchi* sp. n. by the larger, more protuberant proximal bulge on the male chelicerae (Fig. 12B).

This species can also be distinguished from other genotyped taxa (see Fig. 3) by the following nine unique nucleotide substitutions for COI and COII (n = 3): A(147), T(204), C(300), C(306), G(495), C(804), G(807), G(1491), A(1548).

##### Description.

*Holotype male*: Total length 2.77; leg I femur 2.00; F1/CL ratio 1.95. Cephalothorax dark reddish-brown; legs tan brown with darker annulations; abdomen mottled grey-brown and beige, with reddish-brown dorsal scute and sclerites (Fig. 12A). Carapace short (CH/CL ratio 1.65); 1.03 long, 1.69 high, 1.00 wide; ‘neck’ 0.56 wide; highest point of pars cephalica (HPC) approaching posterior third of ‘head’ (ratio of HPC to post-ocular length 0.62), carapace with pronounced concave depression anterior to HPC; ‘head’ not strongly elevated dorsally (post-ocular ratio 0.21) (Fig. 8H). Chelicerae with proximal tuft and additional comb of accessory setae on anterior face of paturon (Fig. 12B). Abdomen 1.64 long, 1.13 wide; almost spherical in lateral profile, without dorsal hump-like tubercles but with highly recumbent mound-like vestiges; dorsal scute fused anteriorly to epigastric sclerites, extending posteriorly to cover nearly anterior two-thirds of dorsal abdomen. Unexpanded pedipalp (Figs 12C–E) pyriform, with broad, distally curved embolus supported by conductor sclerites 1–2; tegular sclerite 1 (TS 1) porrect, strongly curved in prolateral view, with flattened, broadly rounded, paddle-shaped apex; TS 2–3 projecting beyond retro-distal rim of tegulum.

*Female*: Unknown.

*Variation*: Males (n = 2): total length 2.77–2.79; carapace length 1.03 (invariable); carapace height 1.69 (invariable); CH/CL ratio 1.64–1.65.

##### Distribution and habitat.

*Zephyrarchaea marki* is known only from Thistle Cove at Cape Le Grand National Park, on the far south-eastern coast of Western Australia (Fig. 22). Specimens have been collected by beating and sifting elevated leaf litter in a dense coastal thicket of *Banksia speciosa*.

##### Conservation status.

This species appears to be a rare short-range endemic taxon (Harvey 2002b), with the single known population in the Cape Le Grand National Park potentially threatened by fire, dieback disease (affecting *Banksia* heathland vegetation) and climate change.

#### Zephyrarchaea robinsi

(Harvey, 2002a) comb. n.

http://species-id.net/wiki/Zephyrarchaea_robinsi

Figs 9G 13 23

Austrarchaea robinsi Harvey, 2002a: 35, figs 1–4.

Eastern Massif Assassin Spider

##### Type material.

Holotype female: Stirling Range National Park, Ellen Peak, Western Australia, Australia, 34°21'20"S, 118°19'45"E, pitfall trap near summit, 28.V.1996, S. Barrett (WAM T42580).

##### Other material examined.

**AUSTRALIA: *Western Australia*: Stirling Range National Park:** Ellen Peak, 34°21'30"S, 118°19'57"E, sifting elevated leaf litter under *Lepidosperma* sedges in montane heathland near summit, 1007 m, 6.XI.2007, M. Rix et al., 2 juveniles (WAM T89558^DNA: EP-40-J/EP-41-J^); south face of Pyungoorup Peak, 34°21'54"S, 118°19'44"E, sifting elevated leaf litter under *Lepidosperma* sedges along shaded creek line near waterfall, 5.VIII.2008, M. Rix, M. Harvey, 1 juvenile (moulted cuticle) (WAM T94090); Bluff Knoll, summit track, 800 m SW. of summit, 34°22'49"S, 118°15'01"E, sifting elevated leaf litter under *Lepidosperma* sedges in montane heathland, 897 m, 20.VI.2010, M. Rix, J.D. Roberts, 1 juvenile (WAM T114032^DNA: BK-149-J^); Bluff Knoll, off summit track, 900 m SW. of summit, 34°22'52"S, 118°15'00"E, sifting elevated leaf litter in eucalypt grove near creek line, 877 m, 20.VI.2010, M. Rix, J.D. Roberts, 1 juvenile (WAM T114031^DNA: BK-148-J^); Bluff Knoll, off summit track, 400 m SW. of summit, 34°22'34"S, 118°15'15"E, sifting elevated leaf litter under *Lepidosperma* sedges in montane heathland, 1065 m, 24.V.2011, M. Rix, M. Harvey, G. Binford, 1 juvenile (WAM T118987).

##### Diagnosis.

**Figure 13.**
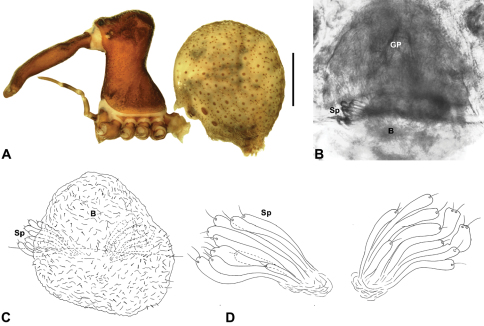
*Zephyrarchaea robinsi* (Harvey, 2002a). **A–D**, Holotype female (WAM T42580) from Ellen Peak, Stirling Range National Park, Western Australia: **A**, cephalothorax and abdomen, lateral view; **B–D**, internal genitalia, dorsal view; **C–D**, illustrations (from Harvey 2002a) of internal genitalia, dorsal view, showing shape of membranous bursa and underlying spermathecae. B = bursa; GP = genital plate; Sp = spermathecae. Scale bar: A = 1.0 mm.<br/>

Females of *Zephyrarchaea robinsi* can be distinguished from *Zephyrarchaea janineae* and *Zephyrarchaea mainae* by the absence of dorsal hump-like tubercles on the abdomen (Fig. 13A); from *Zephyrarchaea austini* sp. n., *Zephyrarchaea grayi* sp. n., *Zephyrarchaea marae* sp. n. and *Zephyrarchaea vichickmani* sp. n. by the shallow post-ocular depression in lateral view (Fig. 9G); and from *Zephyrarchaea barrettae* sp. n. and *Zephyrarchaea melindae* sp. n. by the much shorter carapace (CH/CL ratio < 1.70) (Figs 7, 13A).

This species can also be distinguished from other genotyped taxa (see Fig. 3) by the following three unique nucleotide substitutions for COI and COII (n = 4): C(162), A(531), G(1442).

##### Description.

*Holotype female*: Total length 3.69; leg I femur 1.97; F1/CL ratio 1.60. Cephalothorax reddish-brown; legs tan brown with darker annulations; abdomen variably beige-grey (Fig. 13A). Carapace short (CH/CL ratio 1.60); 1.23 long, 1.97 high, 1.13 wide; ‘neck’ 0.74 wide; highest point of pars cephalica (HPC) approaching posterior third of ‘head’ (ratio of HPC to post-ocular length 0.61), carapace with shallow concave depression anterior to HPC; ‘head’ not strongly elevated dorsally (post-ocular ratio 0.24) (Fig. 9G). Chelicerae without accessory setae on anterior face of paturon. Abdomen 2.10 long, 1.78 wide; spherical in lateral profile, without dorsal hump-like tubercles. Internal genitalia (Figs 13B–D) with cluster of ≤ 15 sausage-shaped spermathecae either side of gonopore, clusters widely separated along midline of genital plate.

*Male*: Unknown.

##### Distribution and habitat.

*Zephyrarchaea robinsi* is known only from Ellen Peak, Bluff Knoll and the south face of Pyungoorup Peak, on the eastern massif of the Stirling Range National Park of southern Western Australia (east of Chester Pass) (Fig. 23). Specimens have been collected by beating and sifting sedges (*Lepidosperma* sp.) in montane heathland habitats and along mesic, shaded creek lines.

##### Conservation status.

This species is a short-range endemic taxon (Harvey 2002b), with a maximum total range of less than 10 km^2^, and all known populations in the eastern Stirling Range National Park **potentially threatened** by fire, dieback disease (affecting montane vegetation) and climate change.

#### Zephyrarchaea melindae

Rix & Harvey sp. n.

urn:lsid:zoobank.org:act:E42EAC7F-09B8-4441-85DD-4D84F44D923C

http://species-id.net/wiki/Zephyrarchaea_melindae

Figs 8E 9H 14 24

Toolbrunup Assassin Spider

##### Type material.

Holotype male: Stirling Range National Park, Mount Hassell, Western Australia, Australia, 34°22'41"S, 118°04'15"E, sifting elevated leaf litter under *Lepidosperma* sedges near summit, 726 m, 22.IV.2009, M. Rix (WAM T118986).

Paratypes: Allotype female and 1 juvenile, Toolbrunup Peak, 34°23'02"S, 118°02'55"E, sifting elevated leaf litter under low herbaceous shrubs near summit, 964 m, 10.IV.2009, M. Rix, H. Wood (WAM T97468^DNA: TP-152-F/TP-153-J^).

##### Other material examined.

**AUSTRALIA: *Western Australia*:** **Stirling Range National Park:** same data as holotype, 2 juveniles (WAM T97467^DNA: HA-150-J/HA-151-J^).

##### Etymology.

The specific epithet is a patronym in honour of Dr Melinda Moir, in recognition of her contributions to biodiversity research, especially in the Stirling Range National Park of southern Western Australia.

##### Diagnosis.

**Figure 14.**
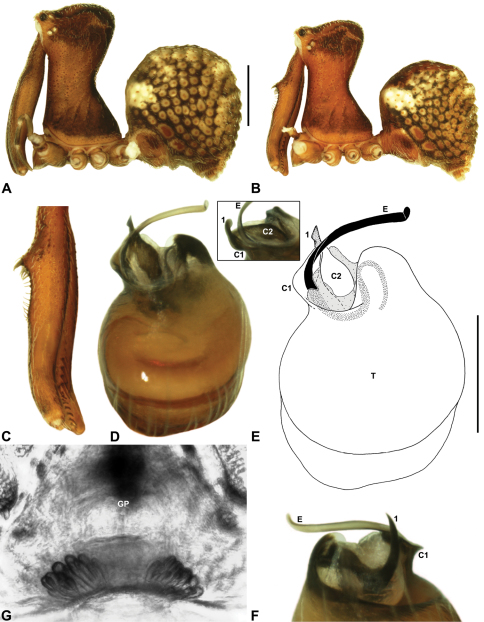
*Zephyrarchaea melindae* sp. n. **A–B**, Cephalothorax and abdomen, lateral view: **A**, allotype female (WAM T97468) from Toolbrunup Peak, Stirling Range National Park, Western Australia; **B**, holotype male (WAM T118986) from Mount Hassell, Stirling Range National Park, Western Australia. **C**, Holotype male chelicerae, lateral view, showing accessory setae. **D–F**, Holotype male pedipalp: **D–E**, bulb, retrolateral view, with inset showing twisted apex of tegular sclerite 1 in retroventral view; **F**, detail of distal tegular sclerites, prolateral view. **G**, Allotype female internal genitalia, dorsal view. C1–2 = conductor sclerites 1–2; E = embolus; GP = genital plate; T = tegulum; (TS)1 = tegular sclerite 1. Scale bars: A–B = 1.0 mm; E = 0.2 mm.<br/>

**Figure 15.**
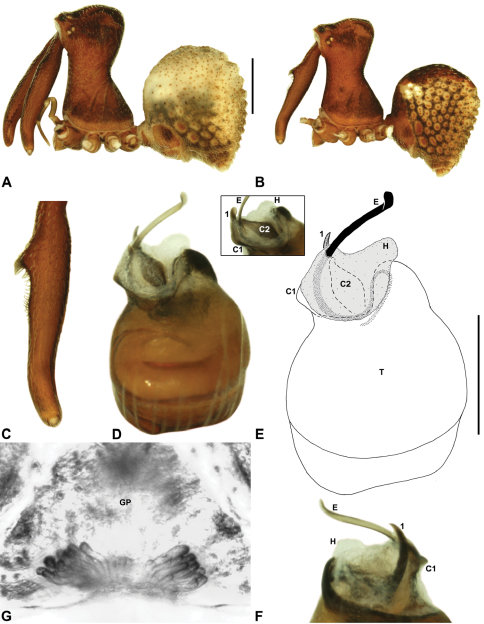
*Zephyrarchaea barrettae* sp. n. **A–B**, Cephalothorax and abdomen, lateral view: **A**, allotype female (WAM T97466) from Talyuberlup Peak, Stirling Range National Park, Western Australia; **B**, holotype male (WAM T117055) from Talyuberlup Peak, Stirling Range National Park, Western Australia. **C**, Holotype male chelicerae, lateral view, showing accessory setae. **D–F**, Holotype male pedipalp: **D–E**, bulb, retrolateral view, with inset showing twisted apex of tegular sclerite 1 in retroventral view; **F**, detail of distal tegular sclerites, prolateral view. **G**, Allotype female internal genitalia, dorsal view. C1–2 = conductor sclerites 1–2; E = embolus; GP = genital plate; H = distal haematodocha; T = tegulum; (TS)1 = tegular sclerite 1. Scale bars: A–B = 1.0 mm; E = 0.2 mm.<br/>

*Zephyrarchaea melindae* can be distinguished from *Zephyrarchaea janineae* and *Zephyrarchaea mainae* by the absence of dorsal hump-like tubercles on the abdomen (Figs 14A–B); from *Zephyrarchaea marae* sp. n., *Zephyrarchaea marki*, *Zephyrarchaea porchi* sp. n. and *Zephyrarchaea vichickmani* sp. n. by the shape of tegular sclerites 2–3, which do not project beyond the retro-distal rim of the tegulum (Figs 14D–E); and from *Zephyrarchaea barrettae* sp. n. by the shape of the anterior margin of the diastema adjacent to the ‘neck’, which is straight (in females) or only slightly concave in lateral view (in males) (Figs 14A–B cf. Figs 15A–B). Females further distinguished from other known congeners by the combination of a spherical abdomen (Fig. 14A), shallow post-ocular depression in lateral view (Fig. 9H), taller carapace (CH/CL ratio > 1.70) (Figs 7, 14A) and straight, almost vertical anterior margin of the diastema adjacent to the ‘neck’ (Fig. 14A).

This species can also be distinguished from other genotyped taxa (see Fig. 3) by the following three unique nucleotide substitutions for COI and COII (n = 4): G(165), G(924), T(1533).

##### Description.

*Holotype male*: Total length 3.15; leg I femur 2.37; F1/CL ratio 2.01. Cephalothorax dark reddish-brown; legs tan brown with darker annulations; abdomen mottled grey-brown and beige, with reddish-brown dorsal scute and sclerites (Fig. 14B). Carapace relatively short (CH/CL ratio 1.77); 1.18 long, 2.09 high, 1.18 wide; ‘neck’ 0.68 wide; highest point of pars cephalica (HPC) approaching posterior third of ‘head’ (ratio of HPC to post-ocular length 0.62), carapace with shallow concave depression anterior to HPC; ‘head’ not strongly elevated dorsally (post-ocular ratio 0.20) (Fig. 8E). Chelicerae with proximal brush and additional comb of accessory setae on anterior face of paturon (Fig. 14C). Abdomen 1.64 long, 1.23 wide; almost spherical in lateral profile, without dorsal hump-like tubercles; dorsal scute fused anteriorly to epigastric sclerites, extending posteriorly to cover anterior two-thirds of dorsal abdomen. Unexpanded pedipalp (Figs 14D–F) pyriform, with broad, distally curved embolus supported by conductor sclerites 1–2; tegular sclerite 1 (TS 1) strongly curved, claw-like in prolateral view, with twisted, flattened and broadly rounded apex; TS 2–3 not projecting beyond retro-distal rim of tegulum.

*Allotype female*: Total length 3.54; leg I femur 2.50; F1/CL ratio 1.93. Cephalothorax dark reddish-brown; legs tan brown with darker annulations; abdomen mottled grey-brown and beige (Fig. 14A). Carapace relatively short (CH/CL ratio 1.86); 1.29 long, 2.41 high, 1.28 wide; ‘neck’ 0.78 wide; highest point of pars cephalica (HPC) approaching middle of ‘head’ (ratio of HPC to post-ocular length 0.57), carapace with shallow concave depression anterior to HPC; ‘head’ not strongly elevated dorsally (post-ocular ratio 0.19) (Fig. 9H). Chelicerae without accessory setae on anterior face of paturon. Abdomen 1.95 long, 1.51 wide; spherical in lateral profile, without dorsal hump-like tubercles. Internal genitalia (Fig. 14G) with cluster of ≤ 15 sausage-shaped spermathecae either side of gonopore, clusters widely separated along midline of genital plate.

##### Distribution and habitat.

*Zephyrarchaea melindae* is known only from the summits of Toolbrunup Peak and nearby Mount Hassell, in the western Stirling Range National Park of southern Western Australia (west of Chester Pass) (Fig. 24). Specimens have been collected by beating and sifting sedges (*Lepidosperma* sp.) and low shrubs in montane heathland habitats.

##### Conservation status.

This species is a short-range endemic taxon (Harvey 2002b), with a maximum total range of less than 10 km^2^, and all known populations in the western Stirling Range National Park potentially threatened by fire, dieback disease (affecting montane vegetation) and climate change.

#### Zephyrarchaea barrettae

Rix & Harvey sp. n.

urn:lsid:zoobank.org:act:D01825F1-C02A-45C9-96F3-0329CA4F1308

http://species-id.net/wiki/Zephyrarchaea_barrettae

Figs 1B 8F 9I 15 25

Talyuberlup Assassin Spider

##### Type material.

**AUSTRALIA:** Holotype male: Stirling Range National Park, Talyuberlup Peak, Western Australia, Australia, 34°24'21"S, 117°57'08"E, sifting elevated leaf litter under *Lepidosperma* sedges near summit, 4.VIII.2008, M. Rix, M. Harvey (WAM T117055^DNA: TA-154-M^).

Paratypes: Allotype female and 2 juveniles, same data as holotype except 8.II.2009, M. Harvey (WAM T97466^DNA: TA-156-J/TA-157-J^).

##### Other material examined.

**AUSTRALIA: *Western Australia*:** **Stirling Range National Park:** same data as holotype, 1 juvenile (WAM T94089^DNA: TA-155-J^); Talyuberlup Peak, 34°24'20"S, 117°57'06"E, sifting elevated and low leaf litter, montane vegetation around rocky peak, 752 m, 12 April 2009, H. Wood, 1♂, 2♀ (CASENT 9028379); same data, 1♀ (CASENT 9034515).

##### Etymology.

The specific epithet is a patronym in honour of Sarah Barrett, for first discovering assassin spiders in the Stirling Range National Park in 1996.

##### Diagnosis.

*Zephyrarchaea barrettae* can be distinguished from *Zephyrarchaea janineae* and *Zephyrarchaea mainae* by the absence of dorsal hump-like tubercles on the abdomen (Figs 15A–B); from *Zephyrarchaea marae* sp. n., *Zephyrarchaea marki*, *Zephyrarchaea porchi* sp. n. and *Zephyrarchaea vichickmani* sp. n. by the shape of tegular sclerites 2–3, which do not project beyond the retro-distal rim of the tegulum (Figs 15D–E); and from *Zephyrarchaea melindae* by the shape of the anterior margin of the diastema adjacent to the ‘neck’, which is slightly (in females) or strongly concave in lateral view (in males) (Figs 15A–B cf. Figs 14A–B). Females further distinguished from other known congeners by the combination of a spherical abdomen (Fig. 15A), shallow post-ocular depression in lateral view (Fig. 9I), taller carapace (CH/CL ratio > 1.70) (Figs 7, 15A) and slightly concave anterior margin of the diastema adjacent to the ‘neck’ (Fig. 15A).

##### Description.

*Holotype male*: Total length 3.13; leg I femur 2.19; F1/CL ratio 1.92. Cephalothorax dark reddish-brown; legs tan brown with darker annulations; abdomen mottled grey-brown and beige, with reddish-brown dorsal scute and sclerites (Fig. 15B). Carapace relatively short (CH/CL ratio 1.71); 1.14 long, 1.95 high, 1.13 wide; ‘neck’ 0.67 wide; highest point of pars cephalica (HPC) approaching posterior third of ‘head’ (ratio of HPC to post-ocular length 0.59), carapace with shallow concave depression anterior to HPC; ‘head’ not strongly elevated dorsally (post-ocular ratio 0.21) (Fig. 8F). Chelicerae with proximal brush and additional comb of accessory setae on anterior face of paturon (Fig. 15C). Abdomen 1.59 long, 1.21 wide; almost spherical in lateral profile, without dorsal hump-like tubercles; dorsal scute fused anteriorly to epigastric sclerites, extending posteriorly to cover anterior two-thirds of dorsal abdomen. Unexpanded pedipalp (Figs 15D–F) pyriform, with broad, distally curved embolus supported by conductor sclerites 1–2; tegular sclerite 1 (TS 1) strongly curved, claw-like in prolateral view, with twisted, flattened and broadly rounded apex; TS 2–3 not projecting beyond retro-distal rim of tegulum.

*Allotype female*: Total length 3.64; leg I femur 2.31; F1/CL ratio 1.77. Cephalothorax dark reddish-brown; legs tan brown with darker annulations; abdomen variably beige-grey (Fig. 15A). Carapace relatively short (CH/CL ratio 1.78); 1.31 long, 2.33 high, 1.26 wide; ‘neck’ 0.78 wide; highest point of pars cephalica (HPC) approaching posterior third of ‘head’ (ratio of HPC to post-ocular length 0.59), carapace with shallow concave depression anterior to HPC; ‘head’ not strongly elevated dorsally (post-ocular ratio 0.22) (Fig. 9I). Chelicerae without accessory setae on anterior face of paturon. Abdomen 1.90 long, 1.69 wide; spherical in lateral profile, without dorsal hump-like tubercles. Internal genitalia (Fig. 15G) with cluster of ≤ 15 sausage-shaped spermathecae either side of gonopore, clusters widely separated along midline of genital plate.

##### Distribution and habitat.

*Zephyrarchaea barrettae* is known only from the summit of Talyuberlup Peak, in the western Stirling Range National Park of southern Western Australia (west of Chester Pass) (Fig. 25). Specimens have been collected by beating and sifting sedges (*Lepidosperma* sp.) in montane heathland.

##### Conservation status.

This species is a short-range endemic taxon (Harvey 2002b), with a maximum total range of less than 10 km^2^, and all known populations in the western Stirling Range National Park potentially threatened by fire, dieback disease (affecting montane vegetation) and climate change.

#### Zephyrarchaea sp. (unidentified juvenile specimens)

##### Note.

In the absence of adult specimens or molecular data, the following juvenile specimens from Western Australia could not be confidently identified as a known species.

##### Material examined.

**AUSTRALIA: *Western Australia*:** **Stirling Range National Park:** Talyuberlup Picnic Area, gully 400 m NW. of carpark, 34°24'42"S, 117°57'12"E, sifting grass patches within gully, 21.VI.2011, D. & S. Harms, 4 juveniles (WAM T118991).

##### Remarks.

These specimens are the first Archaeidae to be collected from lowland habitats in the Stirling Range National Park, and the first members of the Western Australian High Rainfall Zone Clade (Fig. 3) to be discovered in the Stirling Range. All four juveniles possess paired dorsal tubercles on the abdomen, clearly aligning them with *Zephyrarchaea mainae* and *Zephyrarchaea janineae*. *Zephyrarchaea mainae* has a known distribution that extends north to the Porongurup National Park (see Fig. 20), and it is possible that the Talyuberlup Picnic Area may represent a northern extension of this range. Adult specimens or sequence data are required to confirm the identification of this population.

### The Victorian fauna

#### Archaea hickmani

Butler, 1929 nomen dubium

Archaea hickmani Butler, 1929: 46, pl. 2, figs 1–5.
Archaea hickmanni Butler: Canals 1934: 5, fig. 3.
Austrarchaea hickmani (Butler): Forster & Platnick, 1984: 23, fig. 69 (figs 40–50 show an unrelated species of *Austrarchaea* from New South Wales; see below).

##### Type material.

Holotype juvenile (not examined): no specific locality, Victoria, Australia, ~1922 (MV K097).

##### Other material examined. 

**AUSTRALIA: *Victoria*:** no specific locality, 1936, C. Oke, 1 juvenile (AMS KS97261).

##### Nomenclatural remarks.

*Archaea hickmani* was first described by Butler (1929) from a juvenile specimen of unspecified providence, labelled and listed by Butler simply as “Victoria”. Forster and Platnick (1984) examined this holotype, stating that it was in rather poor condition, and noting that a second adult female (labelled as a “homotype”) accompanied the specimen, the latter apparently collected after the original description in 1929. The genitalia of this adult female specimen were illustrated in Forster and Platnick (1984, fig. 69), the specimen was briefly described, and the species was transferred to the genus *Austrarchaea*. Forster and Platnick (1984, figs 40–50) also presented scanning electron micrographs of a juvenile archaeid from near Sydney, erroneously regarded as being conspecific or very closely related to *Austrarchaea hickmani* based on the absence of setose tubercles on the carapace. However, this specimen is clearly a juvenile of an unrelated species of *Austrarchaea*, as evidenced by the abdominal tubercles (see Forster and Platnick 1984, fig. 40) and New South Wales distribution. The Australian Museum collection also has an additional juvenile specimen of *Austrarchaea hickmani* collected by C. Oke in 1936, similarly labelled as being from “Victoria”.

Based on the three known Victorian specimens identified by Butler as *Archaea hickmani*, the species is clearly congeneric and probably even conspecific with one of the four new Victorian species of *Zephyrarchaea* described in this paper. Unfortunately, given the unspecified collection locality of all three specimens, and thus the inability to unequivocally link the single adult female to the holotype or the type locality, this species must be regarded as a nomen dubium.

#### Zephyrarchaea vichickmani

Rix & Harvey sp. n.

urn:lsid:zoobank.org:act:ADCE3562-8F83-49BE-BB5F-F794E7FECB41

http://species-id.net/wiki/Zephyrarchaea_vichickmani

Figs 1A 8B 9A 16 26

Central Highlands Assassin Spider

##### Type material.

Holotype male: Yarra Ranges National Park, Acheron Gap, Victoria, Australia, 37°40'37"S, 145°44'24"E, sifting elevated leaf litter under tree ferns, *Nothofagus* rainforest, 769 m, 29.III.2010, M. Rix (MV K11578).

Paratypes: Allotype female, same data as holotype (MV K11579); 1 female and 6 juveniles, same data as holotype (WAM T112583^DNA: Ar14–49-F/Ar14–133-J/Ar14–134-J^).

##### Other material examined.

**AUSTRALIA: *Victoria*:** **Yarra Ranges National Park:** Acheron Gap, Central Highlands, 6 km NE. of Mount Donna Buang, 37°40'43"S, 145°44'20"E, pitfall trap, *Nothofagus cunninghamii* forest, 25.VI.–29.VIII.1996, G. Milledge, 1♀ (MV K5919); same data except 21.II.–23.IV.1996, 1 juvenile (MV K5920).

##### Etymology.

The specific epithet is a patronym in honour of the late Professor Victor Hickman, for his extraordinary contributions to arachnology and in honour of L. S. Butler’s original (1929) patronym.

##### Diagnosis.

**Figure 16.**
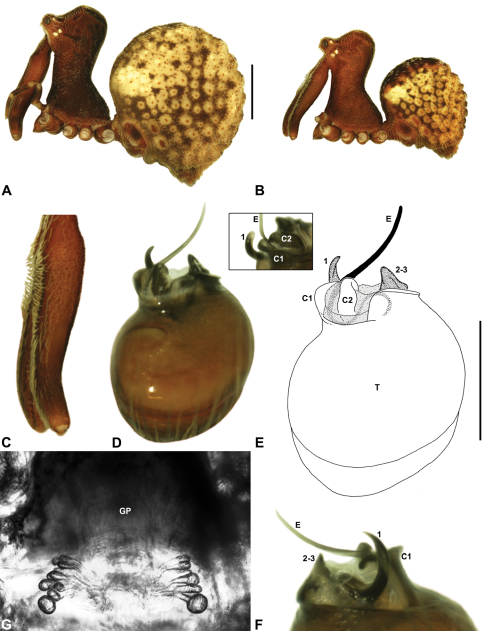
*Zephyrarchaea vichickmani* sp. n. **A–B**, Cephalothorax and abdomen, lateral view: **A**, allotype female (MV K11579) from Acheron Gap, Victoria; **B**, holotype male (MV K11578) from Acheron Gap, Victoria. **C**, Holotype male chelicerae, lateral view, showing accessory setae. **D–F**, Holotype male pedipalp: **D–E**, bulb, retrolateral view, with inset showing twisted apex of tegular sclerite 1 in retroventral view; **F**, detail of distal tegular sclerites, prolateral view. **G**, Allotype female internal genitalia, dorsal view. C1–2 = conductor sclerites 1–2; E = embolus; GP = genital plate; T = tegulum; (TS)1–3 = tegular sclerites 1–3. Scale bars: A–B = 1.0 mm; E = 0.2 mm.<br/>

*Zephyrarchaea vichickmani* can be distinguished from other known congeners except *Zephyrarchaea marae* sp. n. by the absence of a proximal tuft or brush on the male chelicerae (Fig. 16C); and from *Zephyrarchaea marae* sp. n. by the less sinuous shape of conductor sclerite 2 (C2) (Figs 16D–E) and the more concave anterior margin of the male diastema adjacent to the ‘neck’ (Fig. 16B). Females further distinguished from other known congeners except *Zephyrarchaea marae* sp. n. by the combination of a spherical abdomen (Fig. 16A), strongly concave post-ocular depression in lateral view (Fig. 9A), and moderately elevated ‘head’ dorsally (post-ocular ratio ≥ 0.25) (Fig. 9A).

This species can also be distinguished from other genotyped taxa (see Fig. 3) by the following 16 unique nucleotide substitutions for COI and COII (n = 3): C(24), G(54), G(216), G(309), A(360), A(393), C(702), G(795), T(951), A(976), A(1059), T(1063), A(1200), G(1281), C(1479), T(1596), and can be further distinguished from all other Australian Archaeidae except *Zephyrarchaea marae* by the absence of a COII amino acid residue at positions 1441–1443.

##### Description.

*Holotype male*: Total length 2.77; leg I femur 1.80; F1/CL ratio 1.67. Cephalothorax dark reddish-brown; legs tan brown with darker annulations; abdomen mottled grey-brown and beige, with reddish-brown dorsal scute and sclerites (Fig. 16B). Carapace relatively short (CH/CL ratio 1.69); 1.08 long, 1.82 high, 1.01 wide; ‘neck’ 0.59 wide; highest point of pars cephalica (HPC) near posterior third of ‘head’ (ratio of HPC to post-ocular length 0.64), carapace with pronounced concave depression anterior to HPC; ‘head’ moderately elevated dorsally (post-ocular ratio 0.28) (Fig. 8B). Chelicerae with short comb of accessory setae on anterior face of paturon (Fig. 16C). Abdomen 1.59 long, 1.10 wide; almost spherical in lateral profile, without dorsal hump-like tubercles; dorsal scute fused anteriorly to epigastric sclerites, extending posteriorly to cover anterior two-thirds of dorsal abdomen. Unexpanded pedipalp (Figs 16D–F) bulbous, almost spherical, with gently curved, tapering embolus supported by conductor sclerites 1–2; tegular sclerite 1 (TS 1) strongly curved, claw-like in prolateral view, with twisted, flattened and rounded apex; TS 2–3 projecting well beyond retro-distal rim of tegulum.

*Allotype female*: Total length 3.90; leg I femur 2.08; F1/CL ratio 1.76. Cephalothorax dark reddish-brown; legs tan brown with darker annulations; abdomen mottled grey-brown and beige (Fig. 16A). Carapace relatively short (CH/CL ratio 1.83); 1.18 long, 2.15 high, 1.12 wide; ‘neck’ 0.69 wide; highest point of pars cephalica (HPC) approaching posterior third of ‘head’ (ratio of HPC to post-ocular length 0.61), carapace with pronounced concave depression anterior to HPC; ‘head’ moderately elevated dorsally (post-ocular ratio 0.26) (Fig. 9A). Chelicerae without accessory setae on anterior face of paturon. Abdomen 2.51 long, 1.92 wide; spherical in lateral profile, without dorsal hump-like tubercles. Internal genitalia (Fig. 16G) with cluster of ≤ 15 sausage-shaped spermathecae fanning out either side of gonopore, clusters widely separated along midline of genital plate; outermost (posterior) spermatheca on each side stalked, distally spherical.

*Variation*: Females (n = 3): total length 3.44–3.90; carapace length 1.14–1.18; carapace height 2.00–2.15; CH/CL ratio 1.75–1.83.

##### Distribution and habitat.

*Zephyrarchaea vichickmani* is known only from temperate *Nothofagus* rainforest habitats in the Victorian Central Highlands, north-east of Melbourne (Fig. 26).

##### Conservation status.

This species has an imperfectly known distribution, and although potentially restricted, the abundance of protected forested habitats near the type locality would suggest that the species is unlikely to be of conservation concern.

#### Zephyrarchaea marae

Rix & Harvey sp. n.

urn:lsid:zoobank.org:act:010F55AE-B08E-4725-8781-5157D69EDCBA

http://species-id.net/wiki/Zephyrarchaea_marae

Figs 4D 5C–D, 5F–G 6B, 6E 8A, 8C 9B 17 27

West Gippsland Assassin Spider

##### Type material.

Holotype male: Tarra-Bulga National Park, Tarra Valley, near Tarra Valley Picnic Area, Victoria, Australia, 38°26'51"S, 146°32'17"E, sifting elevated leaf litter under tree ferns, *Nothofagus* rainforest, 368 m, 1.IV.2010, M. Rix, D. Harms (MV K11580^DNA: Ar18–138-M^).

Paratypes: Allotype female, Gunyah Rainforest State Reserve, Toorah Road, 2 km SSW. of Gunyah, Victoria, Australia, 38°32'30"S, 146°19'00"E, pitfall trap, *Nothofagus cunninghamii* forest, 14.IX.–14.XI.1995, G. Milledge (MV K5921); 1 male, same data except 5.III.–7.V.1996 (MV K5923).

##### Other material examined.

**AUSTRALIA: *Victoria*:** **Tarra-Bulga National Park:** same data as holotype, 3 juveniles (WAM T114025^DNA: Ar18–139-J/Ar18–140-J^); 0.2 km W. of Tarra Valley Picnic Area, 38°27'S, 146°32'E, pitfall trap, *Nothofagus cunninghamii* forest, 14.XI.1995 – 10.I.1996, G. Milledge, 1 juvenile (MV K5922). **Dandenong Ranges National Park:** Sherbrooke Forest, near start of Welch Track, off Nation Road, 37°54'24"S, 145°22'10"E, sifting elevated leaf litter under tree ferns, wet Mountain Ash/tree fern forest, 28.III.2010, M. Rix, D. Harms, 1♀, 2 juveniles (WAM T114024^DNA: Ar13–135-F/Ar13–136-J/Ar13–137-J^). **Mount Worth State Park:** Giants Circuit from Moonlight Creek Picnic Area, 38°16'54"S, 146°00'35"E, sifting elevated leaf litter under tree fern, complex eucalypt/tree fern forest with thick understorey, 400 m, 31.III.2010, M. Rix, D. Harms, 1 juvenile (WAM T114026^DNA: Ar16–141-J^).

##### Etymology.

The specific epithet is a patronym in honour of Dr Māra Blosfelds, in recognition of her love for small spiders and the Australian forests.

##### Diagnosis.

**Figure 17.**
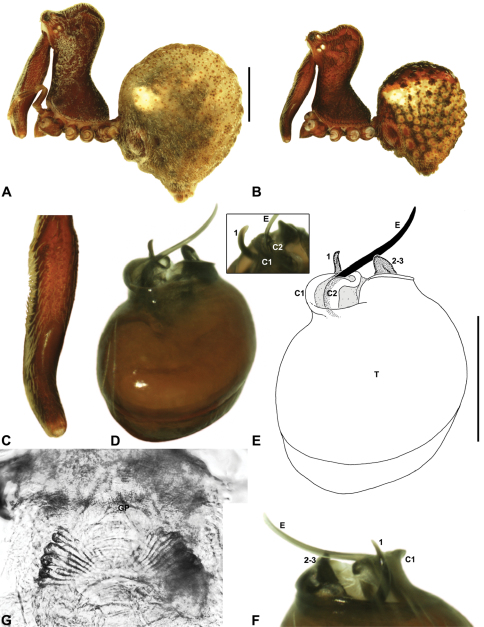
*Zephyrarchaea marae* sp. n. **A–B**, Cephalothorax and abdomen, lateral view: **A**, allotype female (MV K5921) from Gunyah Rainforest State Reserve, Victoria; **B**, holotype male (MV K11580) from Tarra-Bulga National Park, Victoria. **C**, Holotype male chelicerae, lateral view, showing accessory setae. **D–F**, Holotype male pedipalp: **D–E**, bulb, retrolateral view, with inset showing twisted apex of tegular sclerite 1 in retroventral view; **F**, detail of distal tegular sclerites, prolateral view. **G**, Allotype female internal genitalia, dorsal view. C1–2 = conductor sclerites 1–2; E = embolus; GP = genital plate; T = tegulum; (TS)1–3 = tegular sclerites 1–3. Scale bars: A–B = 1.0 mm; E = 0.2 mm.<br/>

*Zephyrarchaea marae* can be distinguished from other known congeners except *Zephyrarchaea vichickmani* by the absence of a proximal tuft or brush on the male chelicerae (Fig. 17C); and from *Zephyrarchaea vichickmani* by the more sinuous, slender, S-shaped conductor sclerite 2 (C2) (Figs 17D–E) and the less concave, slightly convex anterior margin of the male diastema adjacent to the ‘neck’ (Fig. 17B). Females further distinguished from other known congeners except *Zephyrarchaea vichickmani* by the combination of a spherical abdomen (Fig. 17A), strongly concave post-ocular depression in lateral view (Fig. 9B), and moderately elevated ‘head’ dorsally (post-ocular ratio ≥ 0.25) (Fig. 9B).

This species can also be distinguished from other genotyped taxa (see Fig. 3) by the following 12 unique nucleotide substitutions for COI and COII (n = 7): G(69), G(345), G(552), G(762), G(786), A(1026), G(1059), T(1263), G(1341), G(1512), C(1584), C(1587), and can be further distinguished from all other Australian Archaeidae except *Zephyrarchaea vichickmani* by the absence of a COII amino acid residue at positions 1441–1443.

##### Description.

*Holotype male*: Total length 3.03; leg I femur 1.99; F1/CL ratio 1.80. Cephalothorax dark reddish-brown; legs tan brown with darker annulations; abdomen mottled grey-brown and beige, with reddish-brown dorsal scute and sclerites (Fig. 17B). Carapace relatively short (CH/CL ratio 1.70); 1.10 long, 1.87 high, 1.08 wide; ‘neck’ 0.61 wide; highest point of pars cephalica (HPC) near posterior third of ‘head’ (ratio of HPC to post-ocular length 0.66), carapace with pronounced concave depression anterior to HPC; ‘head’ moderately elevated dorsally (post-ocular ratio 0.25) (Figs 8A, 8C). Chelicerae with short comb of accessory setae on anterior face of paturon (Fig. 17C). Abdomen 1.64 long, 1.31 wide; almost spherical in lateral profile, without dorsal hump-like tubercles; dorsal scute fused anteriorly to epigastric sclerites, extending posteriorly to cover anterior two-thirds of dorsal abdomen. Unexpanded pedipalp (Figs 17D–F) bulbous, almost spherical, with gently curved, tapering embolus supported by conductor sclerites 1–2 (C1–2); C2 relatively slender, sinuous, with S-shaped proximal portion; tegular sclerite 1 (TS 1) strongly curved, claw-like in prolateral view, with twisted, flattened and rounded apex; TS 2–3 projecting well beyond retro-distal rim of tegulum.

*Allotype female*: Total length 3.95; leg I femur 2.10; F1/CL ratio 1.76. Cephalothorax dark reddish-brown (with paler, partially encrusted material on ‘neck’); legs tan brown with darker annulations; abdomen variably beige-grey (Fig. 17A). Carapace relatively short (CH/CL ratio 1.86); 1.19 long, 2.22 high, 1.17 wide; ‘neck’ 0.72 wide; highest point of pars cephalica (HPC) approaching posterior third of ‘head’ (ratio of HPC to post-ocular length 0.62), carapace with pronounced concave depression anterior to HPC; ‘head’ moderately elevated dorsally (post-ocular ratio 0.26) (Fig. 9B). Chelicerae without accessory setae on anterior face of paturon. Abdomen 2.31 long, 1.90 wide; spherical in lateral profile, without dorsal hump-like tubercles. Internal genitalia (Fig. 17G) with cluster of ≤ 15 sausage-shaped spermathecae fanning out either side of gonopore, clusters widely separated along midline of genital plate.

*Variation*: Males (n = 2): total length 3.03–3.23; carapace length 1.09–1.10; carapace height 1.85–1.87; CH/CL ratio 1.69–1.70. Females (n = 2): total length 3.74–3.95; carapace length 1.18–1.19; carapace height 2.09–2.22; CH/CL ratio 1.77–1.86.

##### Distribution and habitat.

*Zephyrarchaea marae* is known only from temperate rainforest and mesic closed forest habitats in the Dandenong and Strzelecki Ranges of West Gippsland, east and south-east of Melbourne, Victoria (Fig. 27).

##### Conservation status.

This specieshas a relatively widespread distribution in several National Parks and State Forests, and is not considered to be of conservation concern.

#### Zephyrarchaea porchi

Rix & Harvey sp. n.

urn:lsid:zoobank.org:act:3005E6AB-024D-4559-BAE7-78E3D47CE5E0

http://species-id.net/wiki/Zephyrarchaea_porchi

Figs 8D 18 28

Otway Range Assassin Spider

##### Type material.

Holotype male: Bimbi Park, 2.2 km N. of Cape Otway Lighthouse, Victoria, Australia, 38°50'13"S, 143°30'55"E, dry pitfall trap, grassy edge of bracken-rich dry sclerophyll forest, 2–5.XI.2011, N. Porch & Deakin University Wildlife Field Studies students (MV K11581).

##### Etymology.

The specific epithet is a patronym in honour of Dr Nicholas Porch, for first discovering this species in the Otway Range.

##### Diagnosis.

**Figure 18.**
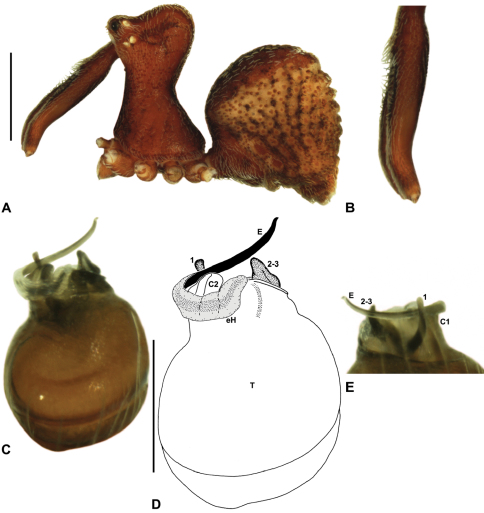
*Zephyrarchaea porchi* sp. n. **A–E**, Holotype male (MV K11581) from Bimbi Park, Otway Range, Victoria: **A**, cephalothorax and abdomen, lateral view; **B**, chelicerae, lateral view, showing accessory setae; **C–D**, pedipalpal bulb (partially expanded), retrolateral view; **E**, detail of distal tegular sclerites, prolateral view. C1–2 = conductor sclerites 1–2; E = embolus; eH = embolic (distal) haematodocha; T = tegulum; (TS)1–3 = tegular sclerites 1–3. Scale bars: A = 1.0 mm; D = 0.2 mm.

*Zephyrarchaea porchi* can be distinguished from *Zephyrarchaea janineae* and *Zephyrarchaea mainae* by the absence of dorsal hump-like tubercles on the abdomen (Fig. 18A); from *Zephyrarchaea marae* and *Zephyrarchaea vichickmani* by the presence of a proximal tuft of accessory setae on the male chelicerae (Fig. 18B); from *Zephyrarchaea barrettae* and *Zephyrarchaea melindae* by the shape of tegular sclerites 2–3, which project well beyond the retro-distal rim of the tegulum (Figs 18C–D); and from *Zephyrarchaea marki* by the smaller, less protuberant proximal bulge on the male chelicerae (Fig. 18B).

##### Description.

*Holotype male*: Total length 2.77; leg I femur 1.97; F1/CL ratio 1.86. Cephalothorax dark reddish-brown; legs tan brown with darker annulations; abdomen mottled grey-brown and beige, with reddish-brown dorsal scute and sclerites (Fig. 18A). Carapace relatively short (CH/CL ratio 1.70); 1.06 long, 1.81 high, 1.00 wide; ‘neck’ 0.58 wide; highest point of pars cephalica (HPC) near posterior third of ‘head’ (ratio of HPC to post-ocular length 0.69), carapace with pronounced concave depression anterior to HPC; ‘head’ moderately elevated dorsally (post-ocular ratio 0.27) (Fig. 8D). Chelicerae with proximal tuft and additional comb of accessory setae on anterior face of paturon (Fig. 18B). Abdomen 1.59 long, 1.08 wide; almost spherical in lateral profile, without dorsal hump-like tubercles; dorsal scute fused anteriorly to epigastric sclerites, extending posteriorly to cover anterior two-thirds of dorsal abdomen. Partially expanded pedipalp (Figs 18C–E) bulbous-pyriform, with gently curved, tapering embolus adjacent to conductor sclerites 1–2; tegular sclerite 1 (TS 1) porrect, slightly curved in prolateral view, with flattened, rounded apex; TS 2–3 projecting well beyond retro-distal rim of tegulum.

*Female*: Unknown.

##### Distribution and habitat.

*Zephyrarchaea porchi* is known only from north of Cape Otway, in the Otway Range of southern Victoria (Fig. 28). The single known specimen was collected in a dry vertebrate pitfall trap in eucalypt forest with a dense bracken fern understorey.

##### Conservation status.

This species has an imperfectly known distribution, and although potentially restricted, the abundance of protected forested habitats near the type locality would suggest that the species is unlikely to be of conservation concern.

#### Zephyrarchaea grayi

Rix & Harvey sp. n.

urn:lsid:zoobank.org:act:B449FA7D-B159-42D5-8F8F-74639C04575E

http://species-id.net/wiki/Zephyrarchaea_grayi

Figs 9C 19A–B 29

Grampians Assassin Spider

##### Type material.

Holotype female: Grampians National Park, Delley’s Dell, Silverband Road, Victoria, Australia, sweeping at night, 26.III.1974, M. Gray (AMS KS109448).

##### Etymology.

The specific epithet is a patronym in honour of Dr Mike Gray, for his contributions to arachnology and for first discovering this species in the Grampians National Park.

##### Diagnosis.

**Figure 19.**
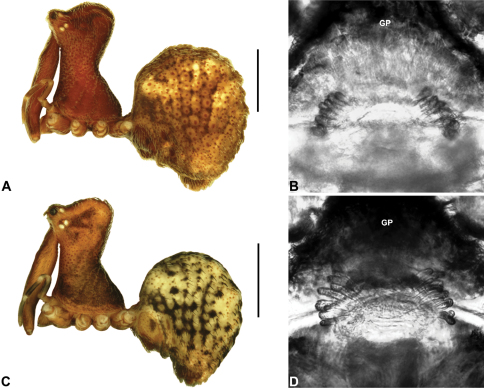
*Zephyrarchaea grayi* sp. n. and *Zephyrarchaea austini* sp. n. **A–B**, holotype female *Zephyrarchaea grayi* sp. n. (AMS KS109448) from Delley’s Dell, Grampians National Park, Victoria: **A**, cephalothorax and abdomen, lateral view; **B**, internal genitalia, antero-dorsal view. **C–D**, holotype female *Zephyrarchaea austini* (SAM NN28000) from Western River Wilderness Protection Area, Kangaroo Island, South Australia: **C**, cephalothorax and abdomen, lateral view; **D**, internal genitalia, antero-dorsal view. GP = genital plate. Scale bars: A, C = 1.0 mm.

**Figure 20.**
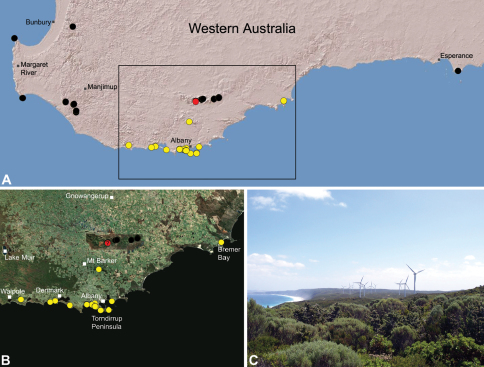
*Zephyrarchaea mainae* (Platnick, 1991b), distribution and habitat: **A**, topographic map showing the known distribution of Archaeidae in south-western Western Australia, with collection localities for *Zephyrarchaea mainae* highlighted in yellow (red highlighted localities denote juvenile specimens of tentative identification; see *Zephyrarchaea* sp. unidentified juvenile specimens, above); **B**, satellite image showing detail of inset (A); **C**, temperate coastal heathland near the type locality – Albany Wind Farm, Torndirrup Peninsula, Western Australia (March 2008). Image (C) by M. Rix.

**Figure 21.**
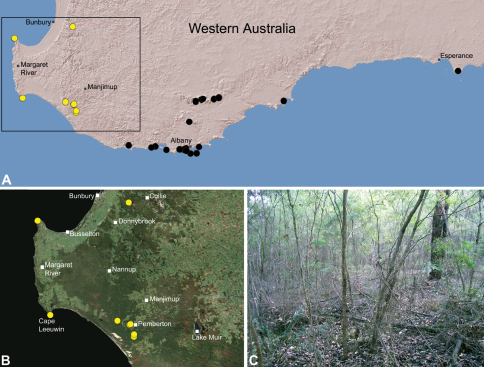
*Zephyrarchaea janineae* sp. n., distribution and habitat: **A**, topographic map showing the known distribution of Archaeidae in south-western Western Australia, with collection localities for *Zephyrarchaea janineae* highlighted in yellow; **B**, satellite image showing detail of inset (A); **C**, wet eucalypt forest at the type locality – Karri Valley, Western Australia (August 2006). Image (C) by M. Rix.

**Figure 22.**
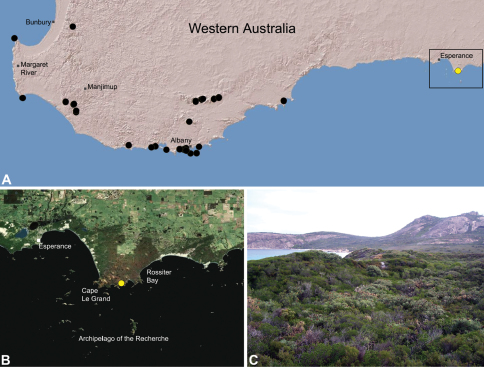
*Zephyrarchaea marki* sp. n., distribution and habitat: **A**, topographic map showing the known distribution of Archaeidae in south-western Western Australia, with collection localities for *Zephyrarchaea marki* highlighted in yellow; **B**, satellite image showing detail of inset (A); **C**, temperate coastal heathland at the type locality – Thistle Cove, Cape Le Grand National Park, Western Australia (June 2010). Image (C) by M. Rix.

**Figure 23.**
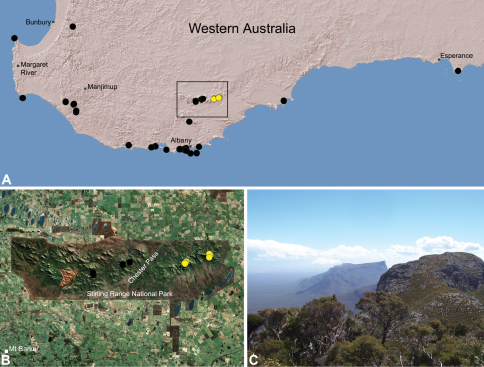
*Zephyrarchaea robinsi* (Harvey, 2002a), distribution and habitat: **A**, topographic map showing the known distribution of Archaeidae in south-western Western Australia, with collection localities for *Zephyrarchaea robinsi* highlighted in yellow; **B**, satellite image showing detail of inset (A); **C**, montane heathland at the type locality – Ellen Peak, Stirling Range National Park, Western Australia (November 2007), with Bluff Knoll visible in the distance. Image (C) by M. Rix.

**Figure 24.**
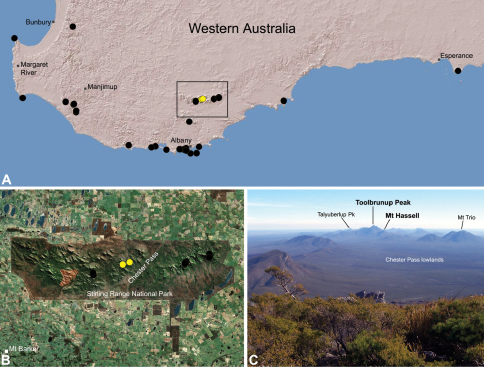
*Zephyrarchaea melindae* sp. n., distribution and habitat: **A**, topographic map showing the known distribution of Archaeidae in south-western Western Australia, with collection localities for *Zephyrarchaea melindae* highlighted in yellow; **B**, satellite image showing detail of inset (A); **C**, view from the summit of Bluff Knoll across the western Stirling Range National Park, showing collection localities for *Zephyrarchaea melindae* (i.e. Toolbrunup Peak, Mount Hassell) highlighted in bold (June 2010). Note the Chester Pass lowlands, separating populations of *Zephyrarchaea melindae* and *Zephyrarchaea robinsi*, and Talyuberlup Peak in the distance, home to *Zephyrarchaea barrettae* sp. n. Image (C) by M. Rix.

**Figure 25.**
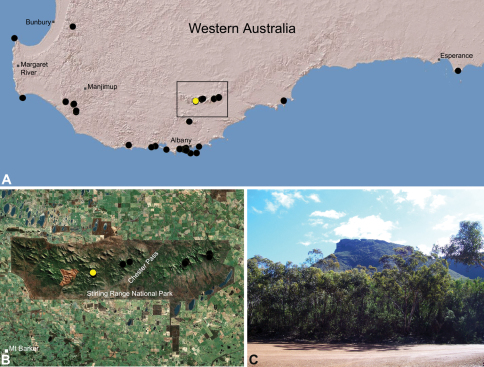
*Zephyrarchaea barrettae* sp. n., distribution and habitat: **A**, topographic map showing the known distribution of Archaeidae in south-western Western Australia, with collection localities for *Zephyrarchaea barrettae* highlighted in yellow; **B**, satellite image showing detail of inset (A); **C**, view from Stirling Range Drive showing the type locality – Talyuberlup Peak, Stirling Range National Park (August 2008). Image (C) by M. Rix.

**Figure 26.**
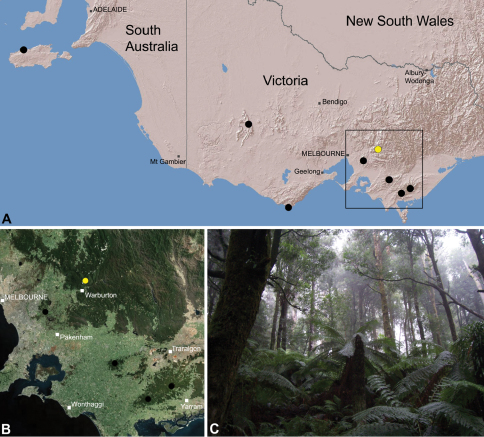
*Zephyrarchaea vichickmani* sp. n., distribution and habitat: **A**, topographic map showing the known distribution of Archaeidae in Victoria and South Australia, with collection localities for *Zephyrarchaea vichickmani* highlighted in yellow; **B**, satellite image showing detail of inset (A); **C**, cool-temperate *Nothofagus* rainforest at the type locality – Acheron Gap, Yarra Ranges National Park, Victoria (March 2010). Image (C) by M. Rix.

**Figure 27.**
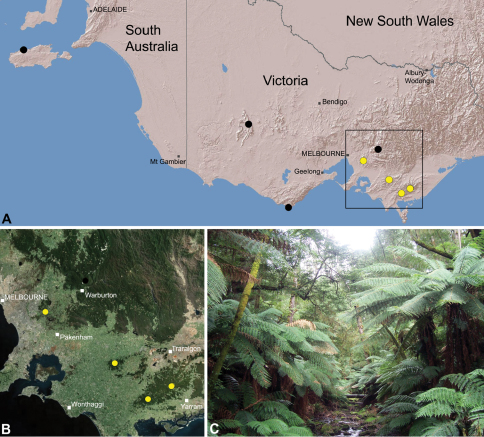
*Zephyrarchaea marae* sp. n., distribution and habitat: **A**, topographic map showing the known distribution of Archaeidae in Victoria and South Australia, with collection localities for *Zephyrarchaea marae* highlighted in yellow; **B**, satellite image showing detail of inset (A); **C**, cool-temperate *Nothofagus* rainforest at the type locality – Tarra Valley, Tarra-Bulga National Park, Victoria (April 2010). Image (C) by M. Rix.

**Figure 28.**
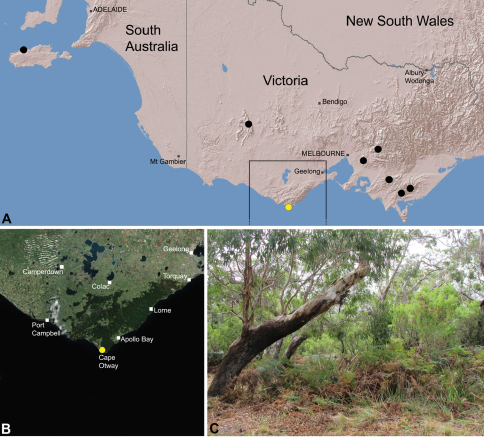
*Zephyrarchaea porchi* sp. n., distribution and habitat: **A**, topographic map showing the known distribution of Archaeidae in Victoria and South Australia, with collection localities for *Zephyrarchaea porchi* highlighted in yellow; **B**, satellite image showing detail of inset (A); **C**, bracken-rich eucalypt forest at the type locality – Bimbi Park, Otway Range, Victoria (March 2012). Image (C) by N. Porch, used with permission.

Females of *Zephyrarchaea grayi* can be distinguished from *Zephyrarchaea janineae* and *Zephyrarchaea mainae* by the absence of dorsal hump-like tubercles on the abdomen (Fig. 19A); from *Zephyrarchaea barrettae*, *Zephyrarchaea melindae* and *Zephyrarchaea robinsi* by the strongly concave post-ocular depression in lateral view (Fig. 9C); from *Zephyrarchaea austini* sp. n. by the larger body size (carapace length > 1.10) and taller carapace (CH/CL ratio ≥ 1.70) (Figs 7, 19A); and from *Zephyrarchaea marae* and *Zephyrarchaea vichickmani* by the shape of the ‘head’, which is less elevated dorsally (post-ocular ratio < 0.25) and with the highest point of the pars cephalica (HPC) closer to the middle of the head (ratio of HPC to post-ocular length < 0.60) (Fig. 9C).

##### Description.

*Holotype female*: Total length 3.36; leg I femur 1.95; F1/CL ratio 1.73. Cephalothorax dark reddish-brown; legs tan brown with darker annulations; abdomen mottled grey-brown and beige (Fig. 19A). Carapace relatively short (CH/CL ratio 1.72); 1.13 long, 1.94 high, 1.03 wide; ‘neck’ 0.63 wide; highest point of pars cephalica (HPC) approaching middle of ‘head’ (ratio of HPC to post-ocular length 0.57), carapace with pronounced concave depression anterior to HPC; ‘head’ not strongly elevated dorsally (post-ocular ratio 0.23) (Fig. 9C). Chelicerae without accessory setae on anterior face of paturon. Abdomen 1.85 long, 1.36 wide; spherical in lateral profile, without dorsal hump-like tubercles. Internal genitalia (Fig. 19B) with cluster of ≤ 15 sausage-shaped spermathecae fanning out either side of gonopore, clusters widely separated along midline of genital plate; outermost (posterior) spermathecae bulbous distally.

*Male*: Unknown.

##### Distribution and habitat.

**Figure 29.**
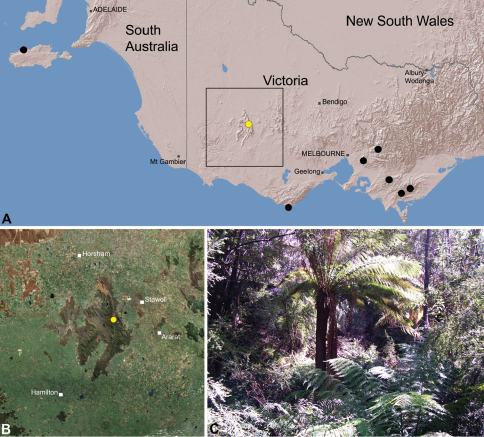
*Zephyrarchaea grayi* sp. n., distribution and habitat: **A**, topographic map showing the known distribution of Archaeidae in Victoria and South Australia, with collection localities for *Zephyrarchaea grayi* highlighted in yellow; **B**, satellite image showing detail of inset (A); **C**, wet sclerophyll forest at the type locality – Delley’s Dell, Grampians National Park, Victoria (March 2010). Image (C) by M. Rix.

*Zephyrarchaea grayi* is known only from wet eucalypt forest at Delley’s Dell, in the Grampians National Park of western Victoria (Fig. 29).

##### Conservation status.

This species appears to be a rare short-range endemic taxon (Harvey 2002b), with the single known population in the Grampians National Park potentially threatened by fire and climate change. Targeted searching at the type locality in March 2010 failed to reveal new specimens of this species, and the site had been recently burnt.

### The South Australian fauna

#### Zephyrarchaea austini

Rix & Harvey sp. n.

urn:lsid:zoobank.org:act:9571EBAF-A9E3-4053-9549-D36E06806DBE

http://species-id.net/wiki/Zephyrarchaea_austini

Figs 9D 19C-D 30

Kangaroo Island Assassin Spider

##### Type material.

Holotype female: Kangaroo Island, Western River Wilderness Protection Area, Waterfall Creek walking trail, near waterfall, South Australia, Australia, 35°41'44"S, 136°54'37"E, sifting elevated leaf litter, open eucalypt woodland with complex understorey of *Xanthorrhoea* and low shrubs, 9-10.V.2010, M. Rix, D. Harms (SAM NN28000^DNA:^ ^Ar77-50-F^).

##### Other material examined.

**AUSTRALIA: *South Australia*:** **Western River Wilderness Protection Area:** same data as holotype, 3 juveniles (WAM T114027^DNA: Ar77-142-J/Ar77-143-J^).

##### Etymology.

The specific epithet is a patronym in honour of Professor Andy Austin, in recognition of his contributions to biodiversity research.

##### Diagnosis.

Females of *Zephyrarchaea austini* can be distinguished from *Zephyrarchaea janineae* and *Zephyrarchaea mainae* by the absence of dorsal hump-like tubercles on the abdomen (Fig. 19C); from *Zephyrarchaea barrettae*, *Zephyrarchaea melindae* and *Zephyrarchaea robinsi* by the strongly concave post-ocular depression in lateral view (Fig. 9D); and from *Zephyrarchaea grayi*, *Zephyrarchaea marae* and *Zephyrarchaea vichickmani* by the smaller body size (carapace length < 1.10) and shorter carapace (CH/CL ratio < 1.70) (Figs 7, 19C).

This species can also be distinguished from other genotyped taxa (see Fig. 3) by the following 45 unique nucleotide substitutions for COI and COII (n = 3): T(42), A(45), C(130), T(132), G(222), A(243), G(291), A(354), G(360), T(372), A(453), A(504), G(567), G(648), A(690), A(702), C(717), C(721), G(756), C(852), A(858), A(891), G(901), A(910), G(962), C(971), G(1050), G(1165), G(1197), A(1198), T(1233), G(1234), C(1235), T(1236), G(1317), A(1374), A(1403), T(1404), G(1416), G(1418), A(1419), A(1449), T(1530), G(1574), G(1585).

##### Description.

*Holotype female*: Total length 2.77; leg I femur 1.80; F1/CL ratio 1.80. Cephalothorax pale reddish-brown; legs tan brown with darker annulations; abdomen mottled grey-brown and beige (Fig. 19C). Carapace short (CH/CL ratio 1.58); 1.00 long, 1.58 high, 0.92 wide; ‘neck’ 0.55 wide; highest point of pars cephalica (HPC) near posterior third of ‘head’ (ratio of HPC to post-ocular length 0.67), carapace with pronounced concave depression anterior to HPC; ‘head’ not strongly elevated dorsally (post-ocular ratio 0.23) (Fig. 9D). Chelicerae without accessory setae on anterior face of paturon. Abdomen 1.49 long, 1.13 wide; almost spherical in lateral profile, without dorsal hump-like tubercles. Internal genitalia (Fig. 19D) with cluster of ≤ 15 sausage-shaped spermathecae fanning out either side of gonopore, clusters widely separated along midline of genital plate.

*Male*: Unknown.

##### Distribution and habitat.

**Figure 30.**
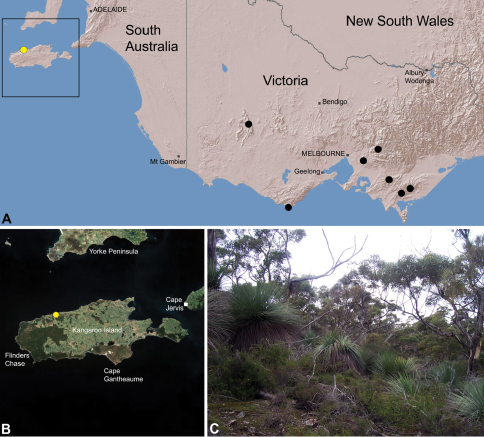
*Zephyrarchaea austini* sp. n., distribution and habitat: **A**, topographic map showing the known distribution of Archaeidae in Victoria and South Australia, with collection localities for *Zephyrarchaea austini* highlighted in yellow; **B**, satellite image showing detail of inset (A); **C**, open eucalypt woodland and heathland at the type locality – near Billy Goat Falls, Western River Wilderness Protection Area, Kangaroo Island, South Australia (May 2010). Image (**C**) by M. Rix.

*Zephyrarchaea austini* is known only from eucalypt woodland and associated heathland habitats near ‘Billy Goat Falls’, in the Western River Wilderness Protection Area of Kangaroo Island, South Australia (Fig. 30).

##### Conservation status.

This species appears to be a rare short-range endemic taxon (Harvey 2002b), with the single known population on Kangaroo Island potentially threatened by fire, dieback disease (affecting heathland vegetation) and climate change.

## Supplementary Material

XML Treatment for Zephyrarchaea

XML Treatment for Zephyrarchaea mainae

XML Treatment for Zephyrarchaea janineae

XML Treatment for Zephyrarchaea marki

XML Treatment for Zephyrarchaea robinsi

XML Treatment for Zephyrarchaea melindae

XML Treatment for Zephyrarchaea barrettae

XML Treatment for Zephyrarchaea

XML Treatment for Archaea hickmani

XML Treatment for Zephyrarchaea vichickmani

XML Treatment for Zephyrarchaea marae

XML Treatment for Zephyrarchaea porchi

XML Treatment for Zephyrarchaea grayi

XML Treatment for Zephyrarchaea austini
